# Supplementary material for: Carotid near-occlusion diagnostics and its consequences: A systematic review
Source: Eur Stroke J. 2025 Jul 15:23969873251355158. Online ahead of print. doi: 10.1177/23969873251355158 (PMC12264249; doi:10.1177/23969873251355158)
Supplement: sj-pdf-1-eso-10.1177_23969873251355158 – Supplemental material for Carotid near-occlusion diagnostics and its consequences: A systematic review [file sj-pdf-1-eso-10.1177_23969873251355158.pdf]

Carotid near-occlusion diagnostics and its consequences: A systematic review
Online supplement

Table of contents
CNO diagnostics results 2
Diagnostic imaging used in studies after 2005 2
Distinguish CNO from conventional stenosis with US 2
Distinguish CNO from occlusion 2
Calculations of the graphical abstract 3
CNO management results 3
Randomized CEA Vs CAS trials that seem to ignore the existence of CNO 3
Hyperperfusion syndrome 3
Unstudied aspects of CNO 4
Discussion 4
CNO diagnostics and definitions 4
CNO diagnostics: What works and what is used 5
Effect of CNO diagnostics on CNO management and missing CNO studies 5
Design issues of recent CNO meta-analyses 6
Evidence to suggest that good CNO diagnostics is rarely used 6
Future studies of CNO management and its diagnostics 7
Suggested standards and improvements in routine practice 7
Miscellaneous management aspects 7
References 8
Supplemental tables 19
Supplemental table 1, main diagnostic studies 19
Supplemental table 2, other diagnostic studies 21
Supplemental table 3, approaches to CNO diagnosis 23
Supplemental table 4, prevalence in patient series 24
Supplemental table 5, prevalence in treatment series 25
Supplemental table 6, reasons for no prevalence assessment 26
Supplemental table 7, management studies with BMT 27
Supplemental table 8, Perioperative risk/long term post-op, study by study 28
Supplemental table 9, Perioperative risk/long term post-op, subtype by subtype 30
Supplemental figures 31
Supplemental figure 1, forest plot 30-day risk with CEA 31
Supplemental figure 2, forest plot 30-day risk with CAS 32
Supplemental figure 3, forest plot long-term post-op with CEA 33
Supplemental figure 4, forest plot long-term post-op with CAS 34
Supplemental figure 5, possible future diagnostic algorithm 35
Supplemental figure 6, typical US-findings in CNO 36
Supplemental figure 7, partial volume effect on CTA 37
Supplemental figure 8, low-flow effects on CTA 38

CNO Diagnostics results

Diagnostic imaging used in studies after 2005

After the NASCET and ECST analysis presented in 2005, there have been 81 original studies that assessed CNO:

- 50 (62%) used CA. 30 did CA as part of CAS [1-30]. 8 where selected series for only those who underwent CA [31-38]. 6 from when CA was still quite common (2005-2011) [39-43], or with data collection during the same period, but later publication (2014) [44]. 6 recent (2020-2024) seemingly consecutive studies of CEA used CA and did not present why this was ethically or clinically reasonable, 5 from China [45-49] and 1 from Turkey [50] – marking a difference in diagnostic approach between countries. Of all CA studies, only one used feature interpretation [6].
- 28 (35%) used CTA. 14 were from a single group that used feature interpretation [51-64]. 3 also used feature interpretation, but from 2 other groups: 1 created CTA criteria [65], 2 were preliminary and updated pathophysiology assessments [66-67]. 1 used measurement criteria [68]. 1 used measurement criteria for full collapse and did not present how those without full collapse were diagnosed [69]. 2 only assessed full collapse [70-71]. 3 assessed only patency [72-74]. 2 were small case series with unclear diagnostics [75-76]. 1 did not present how CNO was separated from conventional stenosis and these seemed to not be well separated [77]. 1 presented new measurement criteria but did not compare to a reference [78].
- 3 (4%) used US. Of the 3 studies that used US, 1 only assessed full collapse [79], one used arbitrary criteria that were likely not specific for CNO [80], one did not define CNO but was conducted in the context of CNO [81].

PC-MRI for CNO diagnostics is thus far only assessed by a single research group [51-52] and, as far as we know, is not implemented in routine practice anywhere.

Distinguish CNO from conventional stenosis with US

CNO often (67%, 105/157) have high flow velocity in the stenosis (similar to conventional stenoses supplemental figure 6A), more often in those without full collapse (86%, 83/97) than those with full collapse (37%, 22/60) [55-56]. Conversely, of stenoses with high flow velocity in the stenosis, 25% (105/423) were CNOs, of which 20% (83/423) were without full collapse and 5% (22/423) were with full collapse [55-56].

Among cases with high flow velocity in the stenosis, the best known parameter for separating CNO from conventional stenosis is distal peak systolic velocity ≤50 cm/s, but this was moderately accurate (63% sensitivity and 94% specificity) [54-56].

A more traditional approach has been to consider a very severe stenosis with low flow velocity in the stenosis (at times difficult to see flow at all) as CNO (supplemental figure 6B). While this is very specific for CNO (≈99%), it has poor sensitivity (15%, 23/157), higher in those with full collapse (30%, 18/60) than those without full collapse (5%, 5/97) [55-56]. Of CNOs with this US finding, 78% (18/23) have full collapse, why US studies using this "low flow velocity in the stenosis"-approach were considered to assess full collapse in the epidemiological assessment.

Distinguish CNO from occlusion

In recent US studies (using only recent US methods) from 4 different laboratories, 17% (51/292) of CNOs were mistaken for occlusion compared to CTA or CA, more often in those with full collapse (34%, 34/100) than those without full collapse (8%, 14/167) [32, 55-56]. None of these studies used contrast-enhanced ultrasound (CEUS). CEUS has been assessed to separate CNO and occlusion once [74], but with limited details on how the images were assessed. However, CEUS has been used to assess patency several times [82-86], but were not included in our search as CNO was not specifically diagnosed (which is a reasonable design when the objective is patency).

When CTAs are assessed in routine practice, CNOs are mistaken for occlusion in 13% (14/104) compared to expert assessment, more often in those with full collapse (26%, 12/47) than those without full collapse (4%, 2/57) [53]. However, there are several non-intuitive aspects of CTA: There are

resolution pitfalls (partial volume effect, supplemental figure 7) and flow effect pitfalls (the CTA exam only shows where contrast has reached at time of image capture, supplemental figure 8). When these pitfalls are accounted for (expert assessment), CTA is 100% accurate for occlusion compared to CA [87].

PC-MRI mistakes 16% (8/49) of CNOs for occlusion, more often in full collapse (62%, 8/13) than without full collapse (0%, 0/36), but this has only been assessed in one study [52]. For PC-MRI, the expected velocities ("velocity encoding") in the blood vessels of interest must be determined before the scan starts. Lower velocity encoding is preferred for venous studies. Thus far, only arterial velocity encoding has been assessed [52].

Calculations of the graphical abstract
The bar to the left is the prevalence of CNO in ≥50% stenosis (30%), summarized in table 2. The sensitivity and specificity that was used to assess the post-test probability were calculated as the mean value of US and CTA. US was considered to be 15% sensitive and 99.6% specific, based on the above-presented traditional approach with a very severe stenosis with low flow velocity in the stenosis [55-56]. Specificity was based on Johansson et al as the cases with conventional stenosis that were actually misdiagnosed as CNO in routine practice, which has otherwise not been presented [56]. Of false negatives, 84% were categorized as conventional stenosis and 16% as occlusions [55-56]. CTA was considered to be 14% sensitive and 99.5% specific, based on the only existing assessment of how CNOs are diagnosed in routine practice, using the mean value of the local and national samples [53]. Of false positives 79% were categorized as conventional stenosis and 21% as occlusions [53]. For simplicity in the figure, the 6 assessments of dissection or thrombosis were excluded when considering the causes of false positives [53]. Thus, the mean value of US and CTA was 14.5% sensitive and 99.55% specific, with 81.5% of false positives being conventional stenosis and 18.5% occlusions.

If this is presented by assessing 1000 representative patients with ≥50% stenosis, 300 will have CNO. Of the CNOs, 44 (300 x 14.5%) will be detected. Of the 256 CNOs missed, 209 (256 x 81.5%) will be categorized as conventional stenosis and 47 (256 x 18.5%) as occlusions. Of the 700 conventional stenoses, 697 (700 x 99.55%) were correctly identified, and 3 were false positive CNOs. Thus, the test outcomes are 47 (44+3) CNOs, 906 (697+209) conventional stenoses and 47 occlusions.

CNO Management results

Randomized CEA Vs CAS trials that seem to ignore the existence of CNO
None of the numerous trials comparing CEA and CAS assessed CNO as a subgroup, even though many CNOs were likely included in these studies. In a recent meta-analysis, 14 trials randomizing between CEA and CAS and presenting data on perioperative risk were listed [88]. None of these 14 studies presented data on CNO specifically. In 9 (64%), CNO (or any of its relevant synonyms) was not mentioned, but many of these likely included CNOs by accepting ultrasound with high stenosis velocity [89-97]. Two (14%) mentioned CNO in selection criteria: One listed "pseudo-occlusion" as a possible reason for excessive CAS risk (exclusion criteria), but not as a formal exclusion criterion by degree of stenosis [98]. One excluded "string sign" as degree of stenosis, but as ultrasound with high stenosis velocity was accepted, it can be understood in retrospect that many CNOs were included [99]. One (7%) had "occlusion or string sign" as the cause of 15 protocol violations, but not as selection criteria, with unclear number of these being "string sign" [100]. Two (14%) were not assessable (one meeting abstract, one in a non-indexed journal) [101-102].

None of the three major randomized trials of asymptomatic carotid stenosis with a conservative arm made any mention of CNO [103-105]. But as they used ultrasound for grading stenoses, many CNOs were likely actually included.

Hyperperfusion syndrome
In 7 studies with good CNO diagnostics, hyperperfusion syndrome (with varying definition) was seen in 3.0% (12/394) of CNO cases and 1.0% (3/315, p=0.067) of conventional stenosis cases [4-6, 36, 45, 61, 66]. In 2 studies with restrictive CNO diagnostics (emphasized delay of contrast arrival [2] and did not acknowledge existence of CNO without full collapse [7]), hyperperfusion syndrome was seen in 17.5% (16/91) of CNO cases vs 0% (0/219, p<0.001) of conventional stenosis. There was no clear

difference between CNO with full collapse (2.6%, 4/152) and CNO without full collapse (3.8%, 6/158, p=0.75), which was only assessed in studies with good CNO diagnostics [4, 6, 36 45, 61].

Unstudied aspects of CNO
No studies have assessed revascularization within 48 hours of last event for CNO with full collapse, treatment of CNO with artery closure, treatment with trans-carotid artery revascularization, or the risk of stroke recurrence with recurrent events despite BMT (a definition used in a recent guideline [106]).

Discussion

CNO diagnostics and definitions
The diagnostic descriptions in many studies were often vague and our approach was to assume "good" diagnostics unless specific criteria were fulfilled. Thus, some studies that fulfilled our definition of "good" diagnostics might not actually have used diagnostics that is similar to that of NASCET/ECST (the idea of "good" diagnostics). Had data that described the included CNOs been more commonly reported, this assessment would likely have been more accurate, why we propose new reporting standards in the main text.

For decades after the first descriptions, the CNO definition was limited to full collapse, then often called "string sign" [106]. However, in NASCET/ECST, the CNO definition was broader and including cases with normal-appearing yet small distal ICA (i.e. without full collapse) as these would also lead to an underestimation of degree of stenosis if the NASCET-percent comparison was used [107-110]. However, subsequent studies, trials and guidelines have had very varying approaches to CNO definition and diagnostics, often clearly not actually applying NASCET grading as it was done in the NASCET/ECST. To describe this, we introduce four groups of understanding of CNO definition and diagnostics:

1. *Ignorance*, where the existence of CNO is ignored. This is easy to fall into, as many are likely taught (and many schematic figures portray) that NASCET grading is done by comparing the stenosis diameter with the diameter well beyond the stenosis. I.e. skipping the first step of NASCET-grading which is to detect CNOs (and only grade remaining cases with percent). This seems to be widespread as a large amount of carotid stenosis articles claiming NASCET grading falls into this category. Illustrative examples include all CEA/CAS randomized trials, asymptomatic stenosis trials and a relevant diagnostic summary from 2006 (discussed further below) [111], and a European US guideline [112].
2. *Full collapse alone*, where all aspects of CNO is assumed to be applicable to CNO with full collapse alone. Many examples were listed in the 2016 diagnostic CNO review [109], highlighting the need to recognize CNO without full collapse. Indeed, the notion of CNO without full collapse was rarely or vaguely presented in non-trial articles before the 2016 review, but this has changed: Of the 79 CNO articles we detected in our updated search, 46 (58%) included the notion of CNO without full collapse, but a notable exception is one recent American guideline which used "string sign" to describe CNO findings [114]. Another notable study is the only carotid stenosis trial beyond NASCET/ECST to include any CNO assessment (ECST-2) that diagnose CNO by low flow velocity in the stenosis according to the diagnostic criteria in the trial protocol [115], hence will almost only detect CNO with full collapse [52-53].
3. *Trying* and *succeeding* (#4), where CNO without full collapse is clearly recognized but how often they are actually detected depends on how good the CNO diagnostics is. Examples of "trying" includes the notion of diagnosing CNO by detecting a small ICA beyond the stenosis on US [116], which was recommended 19 years based on 2 CNO cases [117] before its sensitivity was more properly assessed (and was quite poor) [32] and its specificity is yet to be assessed. Also, a recent European guideline [106]: It presents the CA interpretive criteria in a way easy to misinterpret that they were used in NASCET/ECST the trials (they were not) and present the Bartlet criteria for grading CNO with CTA even though these have modest diagnostic performance when used blinded. But foremost, this European guideline accepts US as sole preoperative exam (as long as it is done twice) [106], based on the above-mentioned 2006 diagnostic summary that does not include CNO [111]. While this is not excellent, it is still the only carotid management guideline to date that even reaches the "trying" level.

Some articles do not fall clearly into any one category, such as a recent European guideline that recognizes the diagnostics issues and therefore provide no management recommendation for CNOs [118], i.e. have no recommendation for 30% of the intended patient group.

CNO diagnostics: What works and what is used

The most used approach in the recent literature is CA based interpretive criteria (detailed in supplement). Four features of CNO are rated as present or absent and a threshold of ≥2 criteria has been established as standard over time. However, these unvalidated criteria were derived from 32 non-representative cases in NASCET/ECST [108], they were not used for the actual trial assessments in NASCET/ECST. Thus, use of CA with these criteria is not a reasonable guarantee that the diagnostics is similar to NASCET/ECST, especially as they require interpretation with no clear borders do not include that the stenosis should be very severe (why they can be false positive due to anatomical variation). This is in contrast to when using CA to grade conventional stenosis, where a degree of stenosis similar to NASCET/ECST is guaranteed as long as the smallest diameter is compared with the distal ICA well beyond the stenosis (not in the bulb region). The key difference being that the instructions for grading with percent are easy and well presented but the instructions for feature interpretation are difficult and not well presented.

Can feature interpretation be used in routine practice? In addition to the issues raised in the main text about issues of training and retention: Performing this type of assessment can likely be psychologically difficult for many assessors as it has a direct impact on management but is without a clear reference. Thus, consistency in application is likely to be worse than the reliability shown in studies. How long training is needed and what reliability can be achieved and sustained has never been assessed. The closest to such as assessment is that one study with two well-aligned experts had better reliability in two series (kappa 0.70-0.80) than when one of these experts was compared to a more recently trained assessor (kappa 0.58) [17, 43].

US cannot distinguish most CNOs from conventional stenosis: Both have high flow velocity in the stenosis. Of stenoses with high flow velocity, there is thus far no accurate way to distinguish CNO from conventional stenosis with US (table 1). This a rather recent (2018) discovery [52], albeit hinted at previously (but with very limited clarification) [116-117]. No US or clinical guideline have been updated with this clinically relevant revelation – US as sole diagnostic modality (as long as it used twice) is still within current recommendations [106, 114].

A threshold-based diagnosis using CTA is not sufficiently accurate for clinical use. The positive predictive value is 78% and negative predictive value is 90% when used in a blind fashion (supplemental table 1). First, the blinded fashion aspect is what mimics clinical routine. In contrast, when the same assessor both assesses the case with feature interpretation and also measure, the (unblinded), the outcome is much better – but not applicable to clinical routine use. Second, if this threshold-based method is used, those categorized as CNO are not CNO 22% of time, so there will be a substantial number of patients with symptomatic conventional stenosis that do not get the revascularization they need. That 10% of conventional stenosis are actually CNOs is a lesser issue as these have a neutral outcome (see below). As a contrast, the corresponding predictive values for PC-MRI are much better (98% and 95%).

While approximately 15% of CNOs are mistaken for occlusion in all modalities, how often occlusions are CNOs cannot be assessed with our search strategy as assessment of patency does not require the use of a CNO synonym. For US there is a recent dedicated analysis [119]. For CTA, this is an issue of expertise (see above). For PC-MRI, the detection threshold for very low flow is quite poor [52].

Effect of CNO diagnostics on CNO management and missing CNO studies

As the results of management studies with good diagnostics are neutral, CEA/CAS should be considered “unnecessary” (not “harmful”). We found no relevant impact on perioperative risk with CNO diagnostics that was not better explained by a moderate risk increase with CEA for CNO with full collapse (and hence a higher risk in studies with “full collapse alone”).

While perioperative outcomes of >1500 CEA/CAS for CNOs have been presented, we found a critical lack of studies of very early (<48 hours after presenting event) CEA/CAS of CNO with full collapse that has never before been presented. While the stroke risk in this group is unclear (table 3), the stroke recurrences that have been reported foremost occur during the first 2 days after presenting event. Hence, treatment must be performed <24 hours, or even more acute, to be effective. Thus, treatment for CNO with full collapse with a decent chance of being effective is yet to be attempted.

Design issues of recent CNO meta-analyses

Four recent meta-analyses have assessed risk with conservative treatment, perioperative risk and/or long-term follow-up after CEA/CAS [120-123]. These analyses included 46 different studies plus 1 conference report between them: Gupta 38 studies [122], Xue 26 studies [120], Antonopoulos 32 studies and 1 conference report [121], and Meershoek 11 studies in an individual patient-data meta-analysis [123]. None of the studies assessed the diagnostics of the underlying studies, why they all included Radak et al [80], all mixed studies of all CNOs with studies of only CNO with full collapse and neither compared CNO with and without full collapse.

- Gupta and Antonopolous assessed risk to end of follow-up but disregarded follow-up time, resulting in an unclear "risk during follow-up" metric (not annual risk or similar). As follow-up time varies significantly between studies, this is a nonsense comparison: A 15% risk over 5 years is not equivalent to 15% over 1 year but was counted as such.
- Xue assessed annual risk for a combined endpoint of ipsilateral stroke, neurological or cardiac death and myocardial infarction – not ipsilateral stroke alone. This was done even when such data were not reported in the underlying studies. When Xue compared long-term effects between conservative treatment and CEA/CAS, the weighting of studies was likely erroneous as the 34 cases from O'Leary et al [113] was weighted similar as the 309 from Radak [80] and 262 from NASCET/ECST [108] from others. This was relevant as Radak (with questionable diagnostics) and O'Leary (9 cases) were the only studies suggesting need of CEA/CAS. These studies accounted for 47% of the analysis, resulting in a higher estimate of need of CEA/CAS than what is reasonable. When assessing annual risk with conservative treatment alone, the same issues existed but aggravated by counting the short-term outcome from Johansson et al [68] in term of annual risk, resulting in a 67% annual risk of stroke.
- Meershoek included Radak et al (which increased the risk with conservative treatment), but not NASCET/ECST (which would have lowered the risk with conservative treatment) due to the individual patient-data meta-analysis design. Long-term follow-up was capped at 1 year and compared treated and untreated in an unorthodox manner: Assessing from presenting event for conservatively managed cases and from CEA/CAS in those that underwent it, i.e. excluding preoperative events for CAS/CEA treated. This study also predated several recent management studies [12, 57, 60]

Evidence to suggest that good CNO diagnostics is rarely used

No clinical or diagnostic guideline have recommendations that are likely to lead to "good" CNO diagnostics [106, 112, 114, 116, 118, 124]. When the shift from CA to non-invasive diagnostics (US and CTA) occurred, this was based on a review [111] where CNO was not even mentioned and only 4 CNO cases were assessed in all of its underlying studies. As far as we know, US is systematically used and cannot detect CNO well (table 1). While CTA can detect CNO, it does not seem to be used that way in routine practice [53]. There is a vast number of carotid stenosis studies that ignore CNO and some only assess full collapse, which reasonably reflects on the CNO diagnostics used. That CNO is often described as rare (or similar) likely reflects on the CNO diagnostics used. And finally, virtually all recent studies with "good" CNO diagnostics are based on CA (which is not commonly used) or from a single expert group using CTA (detailed in supplement). I.e. if "good" CNO diagnostics is widely available with commonly used methods and management guidelines are adhered to, why is no-one reporting on the controversial issue of stroke risk for conservative treatment in symptomatic CNO? The only study that has presented long-term stroke outcomes when managing symptomatic CNO conservatively used CA [34].

Future studies of CNO management and its diagnostics

Available evidence suggests conservative treatment for symptomatic CNOs, especially those without full collapse. However, there are issues with the available evidence: There were several cross-overs during follow-up in NASCET (51%), less so in ECST (4%), so a benefit might have been missed [108]. The non-existent benefit was an unexpected finding in NASCET/ECST but this was recently confirmed in Garcia-Pastor et al, but that was a non-randomized study [34]. As both NASCET/ECST and Garcia-Pastor used CA to diagnose CNO [34, 108], no study with reproducible diagnostics feasible for routine use has assessed long-term prognosis of conservatively treated symptomatic CNO. Thus, further studies of CNO management are warranted. For such studies to be relevant, the CNO diagnostics must be reproducible, accurate compared to NASCET/ECST diagnostics and preferably have a sliding scale for subgroup analyses. Only PC-MRI fulfils all these criteria.

Future PC-MRI-based studies about CNO management could, at least as a start, take the form of an international registry. If so, it will foremost be the systematic use of conservative management for symptomatic CNO that should be studied. Hence, for such a registry to work, it will not only need the introduction of PC-MRI into centers that currently lack it, but also the willingness to trust its diagnosis and manage the patients accordingly. This registry does not have to include many centers to be viable, as CNOs are common. I.e. it would perhaps rather be a collaboration between early adopters. A registry using current diagnostics and/or management is unlikely to provide any relevant benefit. While CTA with feature interpretation as diagnostic basis is reasonable to achieve between a few collaborating centers, it will not solve the issue of reproducibility of the results in routine practice.

While recent guidelines classified conservative treatment for CNO as level A1 [106], there is still sufficient equipoise for a randomized design to be ethical. Especially so as CEA/CAS is at worst expected to be unnecessary, not harmful, making it more acceptable to randomize to CEA/CAS. Hence, a randomized trial comparing CEA/CAS+BMT Vs BMT alone for PC-MRI diagnosed symptomatic CNO is warranted. Especially so if a registry is difficult to achieve or its results (if positive) does not convince the field to change due to its moderate evidence value.

Addressing possible treatments for CNO with full collapse is also reasonable. As not a single case of treatment with relevant timing has thus far been presented, this issue is not ready for large scale studies.

Suggested standards and improvements in routine practice

CNO should be understood to exist as a degree of stenosis, is common, does not have a percentage degree and should (at least for now) be managed conservatively (even when symptomatic). Two similarly accurate diagnostic solutions are CTA with feature interpretation (which is not very feasible) and PC-MRI (which is not very available). If using feature interpretation, it is reasonable to be guided by the knowledge derived from measurement criteria as described in Holmgren et al [52]. This should not be confused with direct adherence to measurement criteria (such as ≥2 of 4 criteria) which has too poor diagnostic accuracy for clinical use [31]. CA is no guarantee of similar CNO diagnostics as in NASCET/ECST, and therefore has no relevant benefits over CTA. US cannot be recommended as sole preoperative modality as it cannot exclude CNO among cases with high flow velocity in the stenosis and most CNOs have this finding.

Miscellaneous management aspects

From the understanding of CNO, the notion of full collapse is relevant, i.e. that it is only a subset of all CNOs. When the existence of CNO without full collapse is understood, defining full collapse might have clinical implications as the prognosis seems to differ. There has thus far only been one attempt to define full collapse, then to best define high early risk among symptomatic CNOs [57]. That study used CTA measurements and flow velocity in the stenosis. It should be noted that the suggested border includes collapses that will not look threadlike on angiography, but a threadlike appearance is not necessarily where the prognosis starts to differ. This threshold has been validated in the sense that when positive, the risk of early stroke recurrence is high, but there were some quite early strokes among those without full collapse as well [60]. The inter-rater reliability of this border was excellent, albeit only assessed in a small sample [60]. Attempts including other metrics, such as clinical metrics, are also warranted, as are analyses for a dose-response assessment withing cases with full collapse:

Will those near the border to without full collapse have different prognosis than those bordering occlusion?

It has been suggested by many that the CNO findings in NASCET and ECST are post-hoc in the sense of being less reliable [4, 15, 33-34, 36-38, 55-56, 69, 76, 80, 121-123, 125-126]. This is reasonably true by the strictest use of the "post-hoc" term. However, it is questionable if this is a fair categorization. Other analyses that were similarly post-hoc have been more fully embraced by the community, such as the notion of treating within 2 weeks of last event and that the relevant border for treatment is >50% (not >30% or >70%) [106]. Separation of CNO from conventional stenosis was not prespecified before the trials started, but the neither were separation of 30-69% into 30-49% and 50-69% nor separation of <31 days from last event to randomization into <2 and 2-4 weeks [127-128]. The trialists themselves did not describe their separation of degree of ipsilateral stenosis as post-hoc, but rather as the core element of the pooled analysis [110]. There were analyses described as post-hoc by the trialists, but CNO was not one of them [127].

References

1 Neves CRB, Casella IB, da Silva ES, et al. Medical Therapy for Asymptomatic Patients and Stent Placement for Symptomatic Patients Presenting with Carotid Artery Near-Occlusion with Full Collapse. *J Vasc Interv Radiol*. 2018;29:998-1005.

2 Ohta T, Nakahara I, Matsumoto S, et al. Prediction of Cerebral Hyperperfusion After Carotid Artery Stenting by Cerebral Angiography and Single-Photon Emission Computed Tomography Without Acetazolamide Challenge. *Neurosurgery*. 2017;81:512-519.

3 Yan Z, Niu G, Zhang B, et al. Early cerebral hemodynamic changes following unilateral carotid artery stenting in patients with different degrees of carotid stenosis. *Quant Imaging Med Surg*. 2023;13:1655-1663.

4 Cay F, Cil BE, Balcı S, et al. Relevance of Distal Arterial Collapse in Stenting of Atherosclerotic Near-Occlusion of the Carotid Artery. *AJNR* 2020;41:1054-1060.

5 İnanç Y, İnanç Y. Carotid Near-Occlusion Stent Experiences. *Noro Psikiyatr Ars.* 2018;57:85-88.

6 Tsai CH, Chen YH, Lin MS, et al. The periprocedural and 30-day outcomes of carotid stenting in patients with carotid artery near-occlusion. *Sci Rep* 2021;11:21876.

7 Zhang L, Dai D, Li Z, et al. Risk factors for hyperperfusion-induced intracranial hemorrhage after carotid artery stenting in patients with symptomatic severe carotid stenosis evaluation. *J Neurointerv Surg*. 2019;11:474-478.

8 Ruiz-Salmerón RJ, Gamero MA, Carrascosa C, et al. Carotid artery stenting: clinical and procedural implications for near-occlusion stenosis. *Neurologia*. 2013;28:535-42.

9 Son S, Choi DS, Kim SK, et al. Carotid artery stenting in patients with near occlusion: a single-center experience and comparison with recent studies. *Clin Neurol Neurosurg*. 2013;115:1976-81.

10 Štěchovský C, Hulíková Tesárková K, Hájek P, et al. Comparison of 30-Day Outcomes after Carotid Artery Stenting in Patients with Near-Occlusion and Severe Stenosis: A Propensity Score Matching Analysis. *AJNR*. 2022;43:1311-1317.

11 Yan D, Tang X, Shi Z, et al. Perioperative and Follow-up Results of Carotid Artery Stenting and Carotid Endarterectomy in Patients with Carotid Near-Occlusion. *Ann Vasc Surg* 2019;59:21-27.

12 Song LP, Zhang WW, Gu YQ, et al. Cognitive improvement after carotid artery stenting in patients with symptomatic internal carotid artery near-occlusion. *J Neurol Sci* 2019;404:86-90.

13 Terada T, Tsuura M, Matsumoto H, et al. Endovascular treatment for pseudo-occlusion of the internal carotid artery. *Neurosurgery.* 2006;59:301-9.

14 Spacek M, Martinkovicova L, Zimolova P, et al. Mid-term outcomes of carotid artery stenting in patients with angiographic string sign. *Catheter Cardiovasc Interv.* 2012;79:174-9.

15 Omoto K, Takayama K, Myouchin K, et al. Carotid Artery Stenting for Near Occlusion with Full Collapse. *World Neurosurg*. 2022;163:e215-e222.

16 Nikas DN, Ghany MA, Stabile E, et al. Carotid artery stenting with proximal cerebral protection for patients with angiographic appearance of string sign. *JACC Cardiovasc Interv*. 2010;3:298-304.

17 González A, Gil-Peralta A, Mayol A, et al. Internal carotid artery stenting in patients with near occlusion: 30-day and long-term outcome. *AJNR*. 2011;32:252-8.

18 Sakamoto S, Kiura Y, Kajihara Y, et al. Carotid artery stenting using the proximal or dual protection method for near occlusion of the cervical internal carotid artery. *Neurosurg Rev.* 2013;36:551-8.

19 Matsuda Y, Terada T, Okada H, et al. Angiographic Characteristics of Pseudo-occlusion of the Internal Carotid Artery Before and After Stenting. *Neurosurgery* 2016;79:832-838.

20 Yan Z, Yang M, Niu G, et al. Cerebral Hemodynamic Variations in the Early Stage after Carotid Artery Stenting in Patients with and without Near Occlusion. *Ann Vasc Surg* 2019;59:5-11.

21 Fanous AA, Natarajan SK, Jowdy PK, et al. High-Risk Factors in Symptomatic Patients Undergoing Carotid Artery Stenting With Distal Protection: Buffalo Risk Assessment Scale (BRASS). *Neurosurgery* 2015;77:531-42; discussion 542-3.

22 Akkan K, Ilgit E, Onal B, et al. Endovascular Treatment for Near Occlusion of the Internal Carotid Artery : 30-Day Outcome and Long-Term Follow-Up. *Clin Neuroradiol.* 2018;28:245-252.

23 Atchaneeyasakul K, Khandelwal P, Ambekar S, et al. Safety Outcomes Using a Proximal Protection Device in Carotid Stenting of Long Carotid Stenoses. *Interv Neurol.* 2016;5:123-130.

24 Mo D, Luo G, Wang B, et al. Staged carotid artery angioplasty and stenting for patients with high-grade carotid stenosis with high risk of developing hyperperfusion injury: a retrospective analysis of 44 cases. *Stroke Vasc Neurol.* 2016;1:147-153.

25 Kang CH, Roh J, Yeom JA, et al. Asymptomatic Cerebral Vasoconstriction after Carotid Artery Stenting. *AJNR*. 2020;41:305-309.

26 Edgell RC, Yavagal DR, Agner C, et al. Recanalization of a symptomatic extracranial internal carotid artery near occlusion with proximal and distal protection: technical case report. *Neurosurgery* 2007;61:E174.

27 Choi BS, Park JW, Shin JE, et al. Outcome evaluation of carotid stenting in high-risk patients with symptomatic carotid near occlusion. *Interv Neuroradiol.* 2010;16:309-16.

28 Barker CM, Gomez J, Grotta JC, et al. Feasibility of carotid artery stenting in patients with angiographic string sign. *Catheter Cardiovasc Interv*. 2010;75:1104-9.

29 Zhang C, Wang Z, Zheng H, et al. Self-expanding stents in the treatment of carotid artery subtotal occlusion: a clinical study on the patients of Hubei and Sichuan in China. *Biomed Mater Eng.* 2012;22:27-33.

30 Oka F, Ishihara H, Kato S, et al. Oka F, Ishihara H, Kato S, Oku T, Yamane A, Kunitugu I, Suzuki M. Cerebral hemodynamic benefits after carotid artery stenting in patients with near occlusion. *J Vasc Surg*. 2013;58:1512-7.

31 Manrique-Zegarra M, García-Pastor A, Castro-Reyes E, et al. CT angiography for diagnosis of carotid near-occlusion: a digital subtraction angiography validation study. *Neuroradiology* 2022;64:1729-1735.

32 Palacios-Mendoza MA, García-Pastor A, Gil-Núñez A, et al. Ultrasonographic and hemodynamic characteristics of patients with symptomatic carotid near-occlusion: results from a multicenter registry study. *Neuroradiology* 2021;63:705-711.

33 García-Pastor A, Gil-Núñez A, Ramírez-Moreno JM, et al. Early risk of recurrent stroke in patients with symptomatic carotid near-occlusion: Results from CAOS, a multicenter registry study. *Int J Stroke* 2017;12:713-719.

34 García-Pastor A, Gil-Núñez A, Ramírez-Moreno JM, et al. The risk of recurrent stroke at 24 months in patients with symptomatic carotid near-occlusion: results from CAOS, a multicentre registry study. *Eur J Neurol*. 2019;26:1391-1398.

35 García-Pastor A, Gil-Núñez A, Ramírez-Moreno JM, et al. Comment on the article "Symptomatic carotid near-occlusion causes a high risk of recurrent ipsilateral ischemic stroke" by Gu et al. *J Neurol*. 2020;267:849-851.

36 Garcia-Pastor A, Gil-Núñez A, Ramirez-Moreno JM, et al. Endarterectomy, Stenting, or Medical Treatment for Symptomatic Carotid Near-Occlusion: Results from CAOS, a Multicenter Registry Study. *AJNR* 2022;43:1304-1310.

37 Kim J, Male S, Damania D, et al. Comparison of Carotid Endarterectomy and Stenting for Symptomatic Internal Carotid Artery Near-Occlusion. *AJNR* 2019;40:1207-1212.

38 García-Pastor A, Gil-Núñez A, Ramírez-Moreno JM, et al. Progression of carotid near-occlusion to complete occlusion: related factors and clinical implications. *J Neurointerv Surg*. 2020;12:1180-1185.

39 Ogata T, Yasaka M, Kanazawa Y, et al. Outcomes associated with carotid pseudo-occlusion. *Cerebrovasc Dis*. 2011;31:494-8.

40 Fujimoto S, Toyoda K, Kishikawa K, et al. Accuracy of conventional plus transoral carotid ultrasonography in distinguishing pseudo-occlusion from total occlusion of the internal carotid artery. *Cerebrovasc Dis*. 2006;22:170-6.

41 Anzidei M, Napoli A, Marincola BC, et al. Gadofosveset-enhanced MR angiography of carotid arteries: does steady-state imaging improve accuracy of first-pass imaging? Comparison with selective digital subtraction angiography. *Radiology*. 2009;251:457-66.

42 Bowman JN, Olin JW, Teodorescu VJ, et al. Carotid artery pseudo-occlusion: does end-diastolic velocity suggest need for treatment?. *Vasc Endovascular Surg*. 2009;43:374-8.

43 Hirata Y, Sakata N, Inoue T, et al. Histopathological features with angiographic correlates of internal carotid artery pseudo-occlusion: impact of plaque compositions. *J Neurosurg.* 2011;115:350-8.

44 Hirata Y, Sakata N, Tsuchimochi H, et al. Carotid Endarterectomy for Pseudo-occlusion of the Cervical Internal Carotid Artery. *Acta Neurochir Suppl*. 2014;119:91-6.

45 Zhang J, Chen J, Xu X, et al. Carotid Endarterectomy for the Treatment of Carotid Near-Occlusion With Recurrent Symptoms. *Front Neurol*. 2022;13:765795.

46 Chai S, Sheng Z, Xie W, et al. Assessment of Apparent Internal Carotid Tandem Occlusion on High-Resolution Vessel Wall Imaging: Comparison with Digital Subtraction Angiography. *AJNR* 2020;41:693-699.

47 Hou Y, Ren L, Cao C, et al. The additional value of high-resolution vessel wall imaging in screening suitable chronic internal carotid artery occlusion candidates for endovascular recanalization: comparison with digital subtraction angiography. Acta Radiol. 2023;64:1702-1711.

48 Wang CM, Han JT, Jia ZC, et al. Hybrid surgery for symptomatic chronic near-total or total occlusion of the internal carotid artery. Chin Med J (Engl). 2021;134:1104-1106.

49 Sun T, Wang C, Han M, et al. Imaging Identification and Prognosis of the Distal Internal Carotid Artery With Near and Complete Occlusion After Recanalization. Front Neurol. 2021;11:630028.

50 İner H, Gökalp O, Yürekli İ, et al. Cartoid Near Occlusion: Time to Re-think Endarectomy?. Anatol J Cardiol. 2024;28:118–23.

51 Johansson E, Zarrinkoob L, Wåhlin A, et al. Diagnosing Carotid Near-Occlusion with Phase-Contrast MRI. *AJNR* 2021;42:927-929.

52 Holmgren M, Henze A, Wåhlin A, et al. Diagnostic separation of conventional ⩾50% carotid stenosis and near-occlusion with phase-contrast MRI. Eur Stroke J. 2024;9:135-143.

53 Johansson E, Gu T, Aviv RI, et al. Carotid near-occlusion is often overlooked when CT angiography is assessed in routine practice. Eur Radiol. 2020;30:2543-2551.

54 Johansson E, Benhabib H, Herod W, et al. Carotid near-occlusion can be identified with ultrasound by low flow velocity distal to the stenosis. Acta Radiol. 2019;60:396-404.

55 Khangure SR, Benhabib H, Machnowska M, et al. Carotid near-occlusion frequently has high peak systolic velocity on Doppler ultrasound. *Neuroradiology* 2018;60:17-25.

56 Johansson E, Vanoli D, Bråten-Johansson I, et al. Near-occlusion is difficult to diagnose with common carotid ultrasound methods. *Neuroradiology* 2021;63:721-730.

57 Johansson E, Gu T, Fox AJ. Defining carotid near-occlusion with full collapse: a pooled analysis. *Neuroradiology* 2022;64:59-67.

58 Gu T, Aviv RI, Fox AJ, et al. Symptomatic carotid near-occlusion causes a high risk of recurrent ipsilateral ischemic stroke. J Neurol. 2020;267:522-530.

59 Johansson E, Fox AJ. Near-Occlusion is a Common Variant of Carotid Stenosis: Study and Systematic Review. Can *J Neurol Sci* 2022;49:55-61.

60 Henze A, Fox AJ, Johansson E. High risk of early recurrent stroke in patients with near-occlusion with full collapse of the internal carotid artery. *Neuroradiology* 2024;66:349-352.

61 Johansson E, Gu T, Castillo S, et al. Intracerebral Haemorrhage after Revascularisation of Carotid Near Occlusion with Full Collapse. *EJVES* 2022;63:523-524.

62 Kellomäki E, Gu T, Fox AJ, et al. Symptomatic and asymptomatic carotid near-occlusions have very similar angiographic appearance on CT-angiography. *Neuroradiology* 2022;64:2203-2206.

63 Johansson E, Aviv RI, Fox AJ. Atherosclerotic ICA stenosis coinciding with ICA asymmetry associated with Circle of Willis variations can mimic near-occlusion. *Neuroradiology* 2020;62:101-104.

64 Holmgren M, Henze A, Wåhlin A, et al. Phase-contrast magnetic resonance imaging of intracranial and extracranial blood flow in carotid near-occlusion. *Neuroradiology* 2024;66:589-599.

65 Bartlett ES, Walters TD, Symons SP, et al. Quantification of carotid stenosis on CT angiography. *AJNR* 2006;27:13-9.

66 Fan X, Lai Z, Lin T, et al. Multidelay MR Arterial Spin Labeling Perfusion Map for the Prediction of Cerebral Hyperperfusion After Carotid Endarterectomy. *J Magn Reson Imaging*. 2023;58:1245-1255.

67 Fan X, Zuo Z, Lin T, et al. Arterial transit artifacts on arterial spin labeling MRI can predict cerebral hyperperfusion after carotid endarterectomy: an initial study. *Eur Radiol*. 2022;32:6145-6157.

68 Johansson E, Öhman K, Wester P. Symptomatic carotid near-occlusion with full collapse might cause a very high risk of stroke. *J Intern Med*. 2015;277:615-23.

69 Pagliariccio G, Di Sario I, Capoccia L, et al. Carotid Near-Occlusion: Surgical or Conservative Management? Retrospective Multicenter Study. *Ann Vasc Surg* 2024;102:133-139.

70 Bennett DL, Hamberg LM, Wang B, et al. Diagnostic yield of delayed phase imaging in CT angiography of the head and neck: a retrospective study. *PLoS One.* 2014;9:e99020.

71 Meershoek AJA, Vonken EPA, Nederkoorn PJ, et al. Carotid endarterectomy in patients with recurrent symptoms associated with an ipsilateral carotid artery near occlusion with full collapse. *J Neurol*. 2018;265:1900-1905.

72 Speranza G, Harish K, Rockman C, et al. Duplex ultrasound and cross-sectional imaging in carotid artery occlusion diagnosis. *J Vasc Surg* 2024;79:577-583.

73 Choi JH, Jang J, Koo J, et al. Multiphasic Computed Tomography Angiography Findings for Identifying Pseudo-Occlusion of the Internal Carotid Artery. *Stroke*. 2020;51:2558-2562.

74 Ventura CA, Silva ES, Cerri GG, et al. Can contrast-enhanced ultrasound with second-generation contrast agents replace computed tomography angiography for distinguishing between occlusion and pseudo-occlusion of the internal carotid artery?. *Clinics (Sao Paulo)*. 2015;70:1-6.

75 Volders D, Shewchuk JR, Marangoni M, et al. Beyond the collaterals: Additional value of multiphase CTA in acute ischemic stroke evaluation. *Neuroradiol J*. 2019;32:309-314.

76 Pisani GP, Calabretto F, Maccario G, et al. Surgery for near occlusion of the internal carotid arteries. A single center experience. *Ann Vasc Surg*. 2021;77:348.

77 Kawabori M, Niiya Y, Iwasaki M, et al. Identification of plaque location using intraoperative indocyanine green during carotid endarterectomy for patient with near occlusion. *J Neurosurg Sci.* 2021;65:397-401.

78 Koskinen SM, Silvennoinen H, Ijäs P, et al. Recognizing subtle near-occlusion in carotid stenosis patients: a computed tomography angiographic study. *Neuroradiology* 2017;59:353-359.

79 Desole A, Campanile F, Tosato F, et al. Surgical treatment for pseudo-occlusion of the internal carotid artery. *Interact Cardiovasc Thorac Surg.* 2015;20:636-40.

80 Radak DJ, Tanaskovic S, Ilijevski NS, et al. Eversion carotid endarterectomy versus best medical treatment in symptomatic patients with near total internal carotid occlusion: a prospective nonrandomized trial. *Ann Vasc Surg* 2010;24:185-9.

81 von Reutern GM, Perren F, Alpsoy I, et al. Poststenotic Distal Caliber Reduction Predicts Very High-Grade Proximal Internal Carotid Artery Stenosis. *Ultraschall Med.* 2023;44:e168-e173.

82 Droste DW, Jürgens R, Nabavi DG, et al. Echocontrast-enhanced ultrasound of extracranial internal carotid artery high-grade stenosis and occlusion. *Stroke.* 1999;30:2302-6.

83 Hammond CJ, McPherson SJ, Patel JV, et al. Assessment of apparent internal carotid occlusion on ultrasound: prospective comparison of contrast-enhanced ultrasound, magnetic resonance angiography and digital subtraction angiography. *EJVES* 2008;35:405-12.

84 Ferrer JM, Samsó JJ, Serrando JR, et al. Use of ultrasound contrast in the diagnosis of carotid artery occlusion. *J Vasc Surg* 2000;31:736-41.

85 Ohm C, Bendick PJ, Monash J, et al. Diagnosis of total internal carotid occlusions with duplex ultrasound and ultrasound contrast. *Vasc Endovascular Surg*. 2005;39:237-43.

86 Hofstee DJ, Hoogland PH, Schimsheimer RJ, et al. Contrast enhanced color duplex for diagnosis of subtotal stenosis or occlusion of the internal carotid artery. *Clin Neurol Neurosurg*. 2000;102:9-12.

87 Chen CJ, Lee TH, Hsu HL, et al. Multi-Slice CT angiography in diagnosing total versus near occlusions of the internal carotid artery: comparison with catheter angiography. *Stroke*. 2004;35:83-5.

88 Batchelder AJ, Saratzis A, Ross Naylor A. Editor's Choice - Overview of Primary and Secondary Analyses From 20 Randomised Controlled Trials Comparing Carotid Artery Stenting With Carotid Endarterectomy. *EJVES* 2019;58:479-493.

89 Naylor AR, Bolia A, Abbott RJ, et al. Randomized study of carotid angioplasty and stenting versus carotid endarterectomy: a stopped trial. *J Vasc Surg* 1998;28:326-34.

90 Brooks WH, Jones MR, Gisler P, et al. Carotid angioplasty with stenting versus endarterectomy: 10-year randomized trial in a community hospital. *JACC Cardiovasc Interv*. 2014;7:163-168.

91 Brooks WH, McClure RR, Jones MR, et al. Carotid angioplasty and stenting versus carotid endarterectomy for treatment of asymptomatic carotid stenosis: a randomized trial in a community hospital. *Neurosurgery* 2004;54:318-24; discussion 324-5.

92 Yadav JS, Wholey MH, Kuntz RE, et al. Protected carotid-artery stenting versus endarterectomy in high-risk patients. *N Engl J Med*. 2004;351:1493-501.

93 Mas JL, Chatellier G, Beyssen B, et al. Endarterectomy versus stenting in patients with symptomatic severe carotid stenosis. *N Engl J Med*. 2006;355:1660-71.

94 SPACE Collaborative Group; Ringleb PA, Allenberg J, Brückmann H, et al. 30 day results from the SPACE trial of stent-protected angioplasty versus carotid endarterectomy in symptomatic patients: a randomised non-inferiority trial. *Lancet*. 2006;368:1239-47.

95 Eckstein HH, Reiff T, Ringleb P, et al. SPACE-2: A Missed Opportunity to Compare Carotid Endarterectomy, Carotid Stenting, and Best Medical Treatment in Patients with Asymptomatic Carotid Stenoses. *EJVES* 2016;51:761-5.

96 Mannheim D, Karmeli R. A prospective randomized trial comparing endarterectomy to stenting in severe asymptomatic carotid stenosis. *J Cardiovasc Surg (Torino).* 2017;58:814-817.

97 Steinbauer MG, Pfister K, Greindl M, et al. Alert for increased long-term follow-up after carotid artery stenting: results of a prospective, randomized, single-center trial of carotid artery stenting vs carotid endarterectomy. *J Vasc Surg* 2008;48:93-8.

98 International Carotid Stenting Study investigators; Ederle J, Dobson J, Featherstone RL, et al. Carotid artery stenting compared with endarterectomy in patients with symptomatic carotid stenosis (International Carotid Stenting Study): an interim analysis of a randomised controlled trial. *Lancet.* 2010;375:985-97.

99 Rosenfield K, Matsumura JS, Chaturvedi S, et al. Randomized Trial of Stent versus Surgery for Asymptomatic Carotid Stenosis. *N Engl J Med.* 2016;374:1011-20.

100 Brott TG, Hobson RW 2nd, Howard G, et al. Stenting versus endarterectomy for treatment of carotid-artery stenosis. *N Engl J Med*. 2010;363:11-23.

101 Alberts MJ. Results of a Multicenter Prospective Randomized Trial of Carotid Artery Stenting vs. Carotid Endarterectomy *Stroke* 2001;32:325

102 Ling F, Jiao LQ. Preliminary report of trial of endarterectomy versus stenting for the treatment of carotid atherosclerotic stenosis in China (TESCAS-C). *Chin J Cerebrovasc Dis* 2006;3:4e8.

103 Endarterectomy for asymptomatic carotid artery stenosis. Executive Committee for the Asymptomatic Carotid Atherosclerosis Study. *JAMA*. 1995;273:1421-8.

104 Halliday A, Mansfield A, Marro J, et al. Prevention of disabling and fatal strokes by successful carotid endarterectomy in patients without recent neurological symptoms: randomised controlled trial. *Lancet.* 2004;363:1491-502.

105 Reiff T, Eckstein HH, Mansmann U, et al. Carotid endarterectomy or stenting or best medical treatment alone for moderate-to-severe asymptomatic carotid artery stenosis: 5-year results of a multicentre, randomised controlled trial. *Lancet Neurol*. 2022;21:877-888.

106 Naylor R, Rantner B, Ancetti S, et al. Editor's Choice - European Society for Vascular Surgery (ESVS) 2023 Clinical Practice Guidelines on the Management of Atherosclerotic Carotid and Vertebral Artery Disease. *EJVES* 2023;65:7-111.

107 Johansson E, Fox AJ. Carotid Near-Occlusion: A Comprehensive Review, Part 1--Definition, Terminology, and Diagnosis. *AJNR* 2016;37:2-10.

108 Fox AJ, Eliasziw M, Rothwell PM, et al. Identification, prognosis, and management of patients with carotid artery near occlusion. *AJNR* 2005;26:2086-94.

109 Johansson E, Fox AJ. Carotid Near-Occlusion: A Comprehensive Review, Part 2--Prognosis and Treatment, Pathophysiology, Confusions, and Areas for Improvement. *AJNR* 2016;37:200-4.

110 Rothwell PM, Eliasziw M, Gutnikov SA, et al. Analysis of pooled data from the randomised controlled trials of endarterectomy for symptomatic carotid stenosis. *Lancet.* 2003;361:107-16.

111 Wardlaw JM, Chappell FM, Best JJ, et al. Non-invasive imaging compared with intra-arterial angiography in the diagnosis of symptomatic carotid stenosis: a meta-analysis. *Lancet.* 2006;367:1503-12.

112 von Reutern GM, Goertler MW, Bornstein NM, et al. Grading carotid stenosis using ultrasonic methods. *Stroke*. 2012;43:916-21.

113 O'Leary DH, Mattle H, Potter JE. Atheromatous pseudo-occlusion of the internal carotid artery. *Stroke.* 1989;20:1168-73.

114 AbuRahma AF, Avgerinos ED, Chang RW, et al. The Society for Vascular Surgery implementation document for management of extracranial cerebrovascular disease. *J Vasc Surg* 2022;75:26S-98S.

115 Cheng SF, van Velzen TJ, Gregson J, et al. The 2nd European Carotid Surgery Trial (ECST-2): rationale and protocol for a randomised clinical trial comparing immediate revascularisation versus optimised medical therapy alone in patients with symptomatic and asymptomatic carotid stenosis at low to intermediate risk of stroke. *Trials.* 2022;23:606.

116 Grant EG, Benson CB, Moneta GL, et al. Carotid artery stenosis: gray-scale and Doppler US diagnosis--Society of Radiologists in Ultrasound Consensus Conference. *Radiology.* 2003;229:340-6.

117 El-Saden SM, Grant EG, Hathout GM, et al. Imaging of the internal carotid artery: the dilemma of total versus near total occlusion. *Radiology.* 2001;221:301-8.

118 Bonati LH, Kakkos S, Berkefeld J, et al. European Stroke Organisation guideline on endarterectomy and stenting for carotid artery stenosis. *Eur Stroke J.* 2021;6:I-XLVII.

119 Rojoa DM, Lodhi AQD, Kontopodis N, et al. Ultrasonography for the diagnosis of extra-cranial carotid occlusion - diagnostic test accuracy meta-analysis. *Vasa*. 2020;49:195-204.

120 Xue S, Tang X, Zhao G, et al. A Systematic Review and Updated Metaanalysis for Carotid Near-Occlusion. *Ann Vasc Surg* 2020;66:636-645.

121 Antonopoulos CN, Giosdekos A, Mylonas SN, et al. Management of internal carotid artery near-occlusion: the need for updated evidence. *Ann Transl Med.* 2020;8:1263.

122 Gupta R, Hassankhani A, Ghozy S, et al. Effect of Treatment Choice on Short-Term and Long-Term Outcomes for Carotid Near-Occlusion: A Meta-Analysis. *World Neurosurg*. 2024;181:e1102-e1129.

123 Meershoek AJA, de Vries EE, Veen D, et al. Meta-analysis of the outcomes of treatment of internal carotid artery near occlusion. *Br J Surg.* 2019;106:665-671.

124 Naylor AR, Ricco JB, de Borst GJ, et al. Editor's Choice - Management of Atherosclerotic Carotid and Vertebral Artery Disease: 2017 Clinical Practice Guidelines of the European Society for Vascular Surgery (ESVS). *EJVES* 2018;55:3-81.

125 García-Pastor A, Iglesias-Mohedano A, Gil-Núñez A. The Impact of Full Collapse on the Risk of Ischaemic Stroke in Patients with Carotid Near Occlusion. *EJVES* 2023;66:4-6.

126 de Borst GJ, Antonopoulos CN, Meershoek AJA, et al. Carotid Artery Near Occlusion: Time to Rethink the Management?. *EJVES* 2020;60:169-170.

127 Rothwell PM, Eliasziw M, Gutnikov SA, et al. Endarterectomy for symptomatic carotid stenosis in relation to clinical subgroups and timing of surgery. *Lancet.* 2004;363:915-24.

128 North American Symptomatic Carotid Endarterectomy Trial Collaborators; Barnett HJM, Taylor DW, Haynes RB, et al. Beneficial effect of carotid endarterectomy in symptomatic patients with high-grade carotid stenosis. *N Engl J Med*. 1991;325:445-53.

129 Rothwell PM, Gutnikov SA, Warlow CP; European Carotid Surgery Trialist's Collaboration. Reanalysis of the final results of the European Carotid Surgery Trial. *Stroke*. 2003;34:514-23.

130 Schulz UG, Rothwell PM. Sex differences in carotid bifurcation anatomy and the distribution of atherosclerotic plaque. *Stroke.* 2001;32:1525-31.

131 Mansour MA, Mattos MA, Hood DB, et al. Detection of total occlusion, string sign, and preocclusive stenosis of the internal carotid artery by color-flow duplex scanning. *Am J Surg.* 1995;170:154-8.

132 Hetzel A, Eckenweber B, Trummer B, et al. Colour-coded duplex sonography of preocclusive carotid stenoses. *Eur J Ultrasound*. 1998;8:183-91.

133 Ringelstein EB, Berg-Dammer E, Zeumer H. The so-called atheromatous pseudoocclusion of the internal carotid artery. A diagnostic and therapeutical challenge. *Neuroradiology.* 1983;25:147-55.

134 Fredericks RK, Thomas TD, Lefkowitz DS, et al. Implications of the angiographic string sign in carotid atherosclerosis. *Stroke.* 1990;21:476-9.

135 Berman SS, Devine JJ, Erdoes LS, et al. Distinguishing carotid artery pseudo-occlusion with color-flow Doppler. *Stroke.* 1995;26:434-8.

136 Lee DH, Gao FQ, Rankin RN, et al. Duplex and color Doppler flow sonography of occlusion and near occlusion of the carotid artery. *AJNR* 1996;17:1267-74.

137 Androulakis AE, Labropoulos N, Allan R, et al. The role of common carotid artery end-diastolic velocity in near total or total internal carotid artery occlusion. *EJVES* 1996;11:140-7.

138 Labropoulos N, Androulakis A, Allan R, et al. The value of colour flow imaging in the detection of subtotal and total internal carotid artery occlusion. *Vasc Surg.* 1997;31:775–779.

139 Fürst G, Saleh A, Wenserski F, et al. Reliability and validity of noninvasive imaging of internal carotid artery pseudo-occlusion. *Stroke.* 1999;30:1444-9.

140 Samson RH, Showalter DP, Yunis JP, et al. Color flow scan diagnosis of the carotid string may prevent unnecessary surgery. *Cardiovasc Surg.* 1999;7:236-41.

141 Leclerc X, Godefroy O, Lucas C, et al. Internal carotid arterial stenosis: CT angiography with volume rendering. *Radiology*. 1999;210:673-82.

142 Lev MH, Romero JM, Goodman DN, et al. Total occlusion versus hairline residual lumen of the internal carotid arteries: accuracy of single section helical CT angiography. *AJNR* 2003;24:1123-9.

143 Ascher E, Markevich N, Hingorani A, et al. Pseudo-occlusions of the internal carotid artery: a rationale for treatment on the basis of a modified carotid duplex scan protocol. *J Vasc Surg* 2002;35:340-5.

144 Johansson E, Holmgren M, Henze A, et al. Diagnosing carotid near-occlusion is a difficult task-but it might get easier. *Neuroradiology* 2022;64:1709-1714.

145 Szabo K, Kern R, Gass A, et al. Acute stroke patterns in patients with internal carotid artery disease: a diffusion-weighted magnetic resonance imaging study. *Stroke.* 2001;32:1323-9.

146 Gabrielsen TO, Seeger JF, Knake JE, et al. The nearly occluded internal carotid artery: a diagnostic trap. *Radiology.* 1981;138:611-8.

147 Greiner C, Wassmann H, Palkovic S, et al. Revascularization procedures in internal carotid artery pseudo-occlusion. *Acta Neurochir (Wien).* 2004;146:237-43.

148 Kniemeyer HW, Aulich A, Schlachetzki F, et al. Pseudo- and segmental occlusion of the internal carotid artery: a new classification, surgical treatment and results. *EJVES* 1996;12:310-20.

149 Sekhar LN, Heros RC, Lotz PR, et al. Atheromatous pseudo-occlusion of the internal carotid artery. *J Neurosurg*. 1980;52:782-9.

150 Regina G, Testini M, Fullone M, et al. Pseudo-occlusion of the internal carotid artery: report of 15 cases and review of the literature. *Int Angiol.* 1997;16:147-50.

151 Remonda L, Heid O, Schroth G. Carotid artery stenosis, occlusion, and pseudo-occlusion: first-pass, gadolinium-enhanced, three-dimensional MR angiography--preliminary study. *Radiology.* 1998;209:95-102.

152 Martin MA, Marotta TR. Vasa vasorum: another cause of the carotid string sign. *AJNR* 1999;20:259-62.

153 Archie JP Jr, Feldtman RW. Critical stenosis of the internal carotid artery. *Surgery.* 1981;89:67-72.

154 Endo S, Hirashima Y, Kurimoto M, et al. Acute pathologic features with angiographic correlates of the nearly or completely occluded lesions of the cervical internal carotid artery. *Surg Neurol.* 1996;46:222-8.

155 Mumenthaler VM, Wellauer J, Schamaun M. "Apoplexie" bei vollständigen und unvollständigen Carotisverschlüssen. *Helv Med Acta*. 1961;28:705-40 contd 808-830.

156 Lippman HH, Sundt TM Jr, Holman CB. The poststenotic carotid slim sign: spurious internal carotid hypolasia. *Mayo Clin Proc.* 1970;45:762-7.

157 Mylonas SN, Antonopoulos CN, Moulakakis KG, et al. Management of Patients with Internal Carotid Artery Near-total Occlusion: An Updated Meta-analysis. *Ann Vasc Surg* 2015;29:1664-72.

158 Johansson E, Fox A. Diagnosing carotid near-occlusion with 1 mm side-to-side asymmetry: a tough task made too easy. *Neuroradiology* 2017;59:319-321.

159 Kargiotis O, Psychogios K, Safouris A, et al. Diagnosis and treatment of acute isolated proximal internal carotid artery occlusions: a narrative review. *Ther Adv Neurol Disord.* 2022;15:17562864221136335.

160 Fisch L, Brown MM. Management of carotid near-occlusion and acute carotid occlusion. *J Cardiovasc Surg (Torino).* 2016;57:145-51.

161 Orrapin S, Rerkasem K. Carotid endarterectomy for symptomatic carotid stenosis. *Cochrane Database Syst Rev*. 2017;6:CD001081.

162 Rerkasem A, Orrapin S, Howard DP, et al. Carotid endarterectomy for symptomatic carotid stenosis. *Cochrane Database Syst Rev*. 2020;9:CD001081.

163 Saba L, Scicolone R, Johansson E, et al. Quantifying Carotid Stenosis: History, Current Applications, Limitations, and Potential: How Imaging Is Changing the Scenario. *Life (Basel).* 2024;14:73.

164 Giannoukas AD, Labropoulos N, Smith FC, et al. Management of the near total internal carotid artery occlusion. *EJVES* 2005;29:250-5.

165 Koutsoumpelis A, Kouvelos G, Peroulis M, et al. Surgical and endovascular intervention on internal carotid artery near occlusion. *Int Angiol.* 2015;34:172-81.

166 Brott TG, Halperin JL, Abbara S, et al. 2011 ASA/ACCF/AHA/AANN/AANS/ACR/ASNR/CNS/SAIP/SCAI/SIR/SNIS/SVM/SVS guideline on the management of patients with extracranial carotid and vertebral artery disease: executive summary: a report of the American College of Cardiology Foundation/American Heart Association Task Force on Practice Guidelines, and the American Stroke Association, American Association of Neuroscience Nurses, American Association of Neurological

Surgeons, American College of Radiology, American Society of Neuroradiology, Congress of Neurological Surgeons, Society of Atherosclerosis Imaging and Prevention, Society for Cardiovascular Angiography and Interventions, Society of Interventional Radiology, Society of NeuroInterventional Surgery, Society for Vascular Medicine, and Society for Vascular Surgery. *J Am Coll Cardiol.* 2011;57:1002-44.

167 Ammar AD, Turrentine MW, Farha SJ. The importance of arteriographic interpretation in occlusion or pseudo-occlusion of the carotid artery. *Surg Gynecol Obstet.* 1988;167:119-23.

168 Berman SS, Bernhard VM, Erly WK, et al. Critical carotid artery stenosis: diagnosis, timing of surgery, and outcome. *J Vasc Surg* 1994;20:499-508; discussion 508-10.

169 Archie JP Jr. Carotid endarterectomy when the distal internal carotid artery is small or poorly visualized. *J Vasc Surg* 1994;19:23-30; discussion 30-1.

170 Pulli R, Frosini P, Gatti M, et al. Internal carotid pseudo-occlusion: Early and late results. *J Cardiovasc Surg (Torino).* 1997;38:15–20.

171 Gil-Peralta A, González A, González-Marcos JR, et al. Internal carotid artery stenting in patients with symptomatic atheromatous pseudo-occlusion. *Cerebrovasc Dis*. 2004;17 Suppl 1:105-12.

172 Henderson RD, Eliasziw M, Fox AJ, et al. Angiographically defined collateral circulation and risk of stroke in patients with severe carotid artery stenosis North American Symptomatic Carotid Endarterectomy Trial (NASCET) Group. *Stroke.* 2000;31:128-32.

173 Rothwell PM, Warlow CP. Low risk of ischemic stroke in patients with reduced internal carotid artery lumen diameter distal to severe symptomatic carotid stenosis: cerebral protection due to low poststenotic flow? On behalf of the European Carotid Surgery Trialists' Collaborative Group. *Stroke.* 2000;31:622-30.

174 Morgenstern LB, Fox AJ, Sharpe BL, et al. The risks and benefits of carotid endarterectomy in patients with near occlusion of the carotid artery North American Symptomatic Carotid Endarterectomy Trial (NASCET) Group. *Neurology.* 1997;48:911-5.

Supplemental table 1. Studies assessing diagnostic accuracy in separating near-occlusion and conventional stenosis and comparing with a reference. All studies used good diagnostics in the reference test

Article	Creates or validates criteria?	Type	Ref	n/N	CNO definition on test	Sens	Spec	PPV[a]	NPV[a]
**CA**									
Fox 2005 [108][b]	Create criteria	IC	FI of same exam	16/32	≥2/4 criteria	91	94	88	95
Rothwell 2000 [129][c]	Validate [130]	MC	FI of same exam	125/3017	ICA/CCA-ratio. Men <0.40, women <0.45	82	99.8	99.5	92
**CTA**									
Bartlett 2006 [65][d]	Create criteria	MC	FI of same exam	42/240	2 selected criteria	92	96	92	96
Johansson 2020 [53]	Validate [65]	MC	FI of same exam	94/358	4/4 criteria	93	97	94	97
Manrique-Zegarra 2022 [31]	Validate [65]	MC	≥2/4 on CA	28/51	≥3/4 criteria	79[e]	89[e]	78[e]	90[e]
Manrique-Zegarra 2022 [31][f]	Create criteria	IC	≥2/4 on CA	28/51	Several	77[e]	83[e]	69[e]	88[e]
**CEMRA or other MRA**									
No studies									
**PC-MRI**									
Johansson 2021 [51]	Create criteria	MC	FI on CTA	9/29	ICA flow ≤110 ml / ICA relative flow ratio <35%	100/100	100/100	100/100	100/100
Holmgren 2024 [52][g]	Validate [51]	MC	FI on CTA	49/239	ICA flow ≤110 ml / ICA relative flow ratio <35%	84/74	98/99	95/ 97	93/89
Holmgren 2024 [52]	Create criteria	MC	FI on CTA	49/239	ICA-CBF flow ratio <22.5%	90	99	98	95
**US**									
Khangure 2018 [55][h]	Validate [131-132]	MC	FI on CTA	54/136	Stenosis PSV <125 cm/s	13	100	100	70
Khangure 2018 [55][h]	Create criteria	MC	FI on CTA	40/118	CCA EDV ≤12 cm/s and/or ICA PSV ≥410 cm/s	98	53	51	98
Johansson 2019 [54]	Create criteria	MC	FI on CTA	27/60	Distal ICA PSV ≤50 cm/s	63	94	84	84
Palacios-Mendoza 2020 [32]	Validate [122]	IC	≥2/4 on CA	135/135	Narrow ICA beyond the stenosis	44	NA	NA	NA
Johansson 2021 [56][i]	Validate [131-132]	MC	FI on CTA	103/445	Stenosis PSV <145 cm/s or otherwise suspected CNO	22	99.3	94	72
Johansson 2021 [56][i]	Create criteria	MC	FI on CTA	65/337	Stenosis EDV≥118 cm/s and/or Stenosis PI ≥2.15	75	75	60	86

CA: Conventional angiography. CCA: Common carotid artery. CEMRA: Contrast-enhanced magnetic resonance angiography. CNO: Carotid near-occlusion. CTA: Computed tomography angiography. EDV: End-diastolic velocity. FI: Feature interpretation. IC: Interpretive criteria. ICA: Internal carotid artery. MC: Measurement criteria. NA: Not available. n/N: number of near-occlusions and total number of observations. NPV: Negative predictive value. PC-MRI: Phase-contrast MRI. PI: Pulsatility index. PSV: Peak systolic velocity. PPV: Positive predictive value. US: Ultrasound

ICA relative flow: ICA of interest / sum of both ICAs. ICA-CBF flow ratio: ICA of interest / sum of both ICAs and Basilar.
Study with duplicate data [68] better presented later [55] not listed.

[a] To enable comparisons, PPV and NPV were calculated from presented sensitivity and specificity, assuming 33% prevalence of CNO (supplemental table 3).
[b] Criteria: Delayed contrast arrival, evidence of collaterals, side-to-side difference in ICA diameter (assessment of ICA ratio) and ICA to ECA diameter comparison (assessment of ECA ratio). No clear border for when a feature is positive. CNO definition: ≥2/4 criteria was the best, other variants also presented.
[c] ICA/CCA ratio was derived from normal-distribution of this metric in cases without stenosis.
[d] Criteria: Stenosis lumen ≤1.3 mm, distal ICA ≤3.5 mm, ICA ratio ≤0.87 and ECA ratio ≤1.27. CNO definition: Combining distal ICA ≤3.5 mm and ICA ratio ≤0.87 was the best, other variants also presented.
[e] 2 observers were presented separately, the average values of these are presented here.
[f] Criteria: Distal ICA diameter smaller than contralateral ICA (assessment of ICA ratio) and distal ICA diameter less or equal to the ipsilateral ECA (assessment of ECA ratio). No clear border for when a feature is positive. Thresholds: 2/2 criteria and the ECA-ratio criteria had same efficacy (presented above), but others also presented.
[g] Criteria: ICA-CBF flow ratio was the best, other variants also presented. When reversing the validation, applying an applicable Holmgren [52] criteria (ICA flow ≤121 ml, 94% sensitive and 96% specific in Holmgren) to Johansson [51] data (possible by a figure in Johansson supplement), Johansson was 100% accurate [51].

[h] Low flow velocity assessed in literature [131-132], but not previously assessed for sensitivity when including CNO without full collapse. When creating criteria, only those with high stenosis velocity (ICA PSV >125 cm/s) were considered. Several criteria and combinations were assessed, CCA EDV and/or ICA PSV was the best.
i Low flow velocity assessed similar to [17] but with slightly higher stenosis PSV (n=10), not certain that there is flow (n=7) and other markers if suggested in routine report (n=6, detailed in article). When creating criteria, only CNOs not already detected were considered. Several criteria and combinations were assessed, the combination of stenosis EDV and stenosis PI was the best.

Supplemental table 2. Other diagnostic studies where at least two approaches are compared.

Article	Year	Reason for not in included in the main diagnostic summary	Numerical main findings, rarely shown to be statistically significant
**US Vs CA (or CTA/MRA when specified)**			
Ringelstein [133]	1983	CNO with full collapse as outcome	Low flow on CW-doppler was always CNO with full collapse, sensitivity not assessed.
Fredricks [134]	1990	CNO with full collapse as outcome	Grey scale was slightly better than CW doppler in detecting flow.
Mansour [131]	1995	CNO with full collapse as outcome	Low flow is very specific for CNO with full collapse, but CNO can have higher flow velocities
Berman [135]	1995	Assess only patency (CNO or occlusion)	Color doppler is better than grey scale
Lee [136]	1996	Assess only patency (CNO or occlusion)	Color doppler is better than grey scale
Androulakis [137]	1996	CNO with full collapse as outcome	CNO with full collapse often have low CCA EDV, similar to occlusions
Labropoulos [138]	1997	Assess only patency (CNO or occlusion)	Color doppler often detects flow
Hetzel [132]	1998	CNO with full collapse as outcome	Low flow is very specific for CNO with full collapse
Fürst [139]	1999	Assess only patency (CNO or occlusion)	Power doppler is better than color doppler. Contrast adds slight sensitivity increase.
Samson [140]	1999	CNO with full collapse alone (no control group)	Patency on color doppler indicates flow on CA.
El-Saden [117]	2001	Assess only patency (CNO or occlusion)	Color doppler often detects flow
Bowman [42]	2009	CNO with full collapse alone (no control group)	Low EDV is common in CNO with full collapse
Ventura [74]	2015	Assess only patency (CNO or occlusion), compares with CTA	Adding contrast clearly increases sensitivity for CNO.
Neves [1]	2018	Assess only patency (CNO or occlusion), no control group, compares with CTA.	Some occlusions on US were patent on CTA
Speranza [72]	2023	Assess only patency (CNO or occlusion) no control group, compares with CTA or MRA.	Some occlusions on US were patent on CTA/MRA
**CTA Vs CA**			
Leclerc [141]	1999	Small sample (n=4 with CNO).	-
Lev [142]	2003	Assess only patency (CNO or occlusion)	Helical CTA often detects flow
Chen [87]	2004	Assess only patency (CNO or occlusion)	Helical CTA often detects flow
**MRA Vs CA**			
Fürst [139]	1999	Assess only patency (CNO or occlusion)	2D and 3D TOF has poor sensitivity, US was better.
El-Saden [117]	2001	Assess only patency (CNO or occlusion)	3D TOF and CEMRA often detects flow
Anzidei [41]	2006	Small sample (n=4 with CNO).	-
Chai [46]	2020	Assess only patency (CNO or occlusion)	High-resolution Vessel-wall imaging MRA showed patency in 6/6 cases occluded on TOF MRA
Hou [47]	2023	Assess only patency (CNO or occlusion)	Considerable overlap with [46]
**Other comparisons**			
Ascher [143]	2002	US Vs previous US or MRA, assess only patency (CNO or occlusion) and no control group	High-sensitive US settings can detect flow in cases where other settings could not.
Fujimoto [40]	2006	US and transoral US Vs CA, assess only patency (CNO or occlusion)	Transoral US detects flow more often than cervical US color doppler
**Non-comparative studies of relevance**			
Grant [116]	2003	Consensus criteria	US consensus criteria, includes CNO criteria of “markedly narrow lumen”[a],
Bennett [70]	2014	Assess patency only, but not specific for CNO.	Presents multiphase CTA for assessing patency. 6/34 occlusions was patent on delayed phase. reproduced in [73, 75].
Schulz [130]	2003	Data not compared with any CNO assessment.	Presents ICA/CCA ratio threshold, based on normal distribution (mean -2 standard deviations), later used to test for CNO [129]
Koskinen [78]	2017	Suggest a novel CNO criteria but with no comparison.	Suggests ICA to ICA side diameter difference of ≥1 mm as CNO definition
Johansson [63]	2019	Presents anatomical variance as differential. Presents a limited assessment of ≥1 mm as CNO definition [98].	Presents an important differential when performing feature interpretation

Johansson [53]	2020	Assess how often CNO are correctly identified in routine practice, i.e. compares assessors.	CNO is often not identified when CTAs are assessed in routine practice.
Johansson [144]	2022	Editorial. Suggests use of post-operative CTA and the highlights then need of clinical validation (stroke risk) of new criteria	Presents post-operative CTA as a new study method.
von Reutern [81]	2022	Only US, compares distal ICA diameter and distal velocity	Confirms the association between low distal velocity on US and small distal ICA on US.

CA: Conventional angiography. CCA: Common carotid artery. CTA: Computed tomography angiography. MRA: Magnetic resonance angiography. CNO: Carotid near-occlusion. ICA: Internal carotid artery. CTA: Computed tomography angiography. US: Ultrasound.

[a] Not specified if in the stenosis or distal to the stenosis, reference to [117] but this study included no clear assessment of narrowing (albeit mentioned) and no control group with conventional stenosis.

Supplemental Table 3. Details about approaches to CNO diagnostics

	Feature interpretation	Measurement criteria	Interpretive criteria
Modalities assessed or often used	CA, CTA	CTA, US, PC-MRI	CA, CTA
Description	Systematic assessment of the whole exam[a].	CTA: 4 measurements[b]<br>US: several velocity measurements<br>PC-MRI: ICA-CBF flow ratio <22.5%	CA: 4 criteria to use combined[c]<br>CTA: 2 criteria to used combined[d]<br>US: 2 criteria suggested.
Main pro	Handles difficult cases[f]	CTA, US: Easy to use<br>PC-MRI: High accuracy and reliability	Quite easy to use, but requires some interpretation
Reliability	Good for collaborating experts[g], but comparisons between studies is difficult.	CTA: Acceptable[g]<br>US: Never assessed<br>PC-MRI: Excellent[g]	Often good[g]
Validation of system	Reference standard [108]	CTA: Not accurate when blindly applied [31]<br>US: Not accurate [54-56]<br>PC-MRI: Very accurate [51-52]	CA: Assessed in 32 cases [108].<br>CTA: Not compared to feature interpretation, and not very accurate [31].<br>US: Not accurate, specificity not assessed [32]
Use in the literature	CA: NASCET+ECST, rarely otherwise<br>CTA: Several recent studies	CTA: Rarely used<br>US: Only a minor part of a trial [115]<br>PC-MRI: Recently proposed, never used	CA: Often used<br>CTA: Recently proposed, never used

CA: Conventional angiography. CAS: Carotid artery stenting. CEMRA: Contrast-enhanced magnetic resonance angiography. CNO: Carotid near-occlusion. CTA: Computed tomography angiography. ICA: Internal carotid artery.

[a] Usually assessed features are stenosis severity, distal ICA diameter (in mm), distal ICA ratio (side-to-side difference), ECA-ratio (ICA-to-ECA difference) and Circle of Willis configuration. Most extensively described in the supplement of Holmgren et al [52]. 5% of cases have too divergent features for a diagnosis to be determined [63] – only PC-MRI has been able to assess such cases well [52].
[c] Delayed time of contrast arrival, evidence of collateral flow, ICA-to-ICA Comparison of Diameter Reduction (i.e. small ICA ratio), ICA-to-ECA Comparison of Diameter Reduction (i.e. small ECA-ratio) [108]. ≥2/4 criteria often used to diagnose CNO.
[d] Distal ICA diameter smaller than contralateral ICA (i.e. small ICA ratio), Distal ICA diameter less than or equal to the ipsilateral ECA (i.e. small ECA ratio) [31].
[b] Stenosis diameter ≤1.3 mm, Distal ICA ≤3.5 mm, ICA-ratio ≤0.87, ECA-ratio ≤1.27 [65]. Number of criteria required is not standardized. Also, a 1-mm ICA-ICA difference has been suggested, but without comparison or later use [78].
[e] First a severe stenosis with low flow velocity. While "low flow velocity" has been arbitrarily assigned in some studies as below the threshold for 50% stenosis, no actual threshold has been developed. Second "markedly narrowed lumen in the distal ICA", which was suggested in a US guideline [116] based on a small study with 2 cases [117] and thus far, only sensitivity has been assessed (and it is quite poor) [32].
[f] The main rationale with systematic interpretation is recognize other causes of small distal ICA (ICA-ratio false positive) than then stenosis, such as anatomical variance. Misleading features includes when both ICAs are small and similar (ICA-ratio false negative) or that CCA stenosis can cause ECA collapse (ECA-ratio false negative). Other easy alternatives are lacking as stenosis severity is reasonably on a spectrum and native distal ICA diameters vary between persons. Also, missing features can be contralateral occlusion or too calcified stenosis to allow for stenosis severity assessment – when so, other features should be more clear for the case to be categorized as CNO.
[g] Inter-rater kappa: Feature interpretation 0.80 [58]. ≥2/4 feature criteria on CA 0.88 [108]. 2/2 feature criteria on CTA 0.80 [31]. Distal ICA ≤3.5 mm 0.31 (and other 3 measurement criteria were worse) [15]. ≥3/4 measurement criteria on CTA 0.31 [15]. PC-MRI: 0.98-1.0 [51-52].

Supplemental table 4 Detailed description of all studies presenting prevalence of CNO in patient series

Denominator	≥90% symptomatic	Study	Year	Symp	Diagnostic approach	Prevalence of CNO % (n/N)
**Good diagnostics**						
≥50% stenosis	Yes	Johansson [68]	2015	100%	CTA+CA FI	31 (20/42)
		Gu [58]	2020	100%	CTA FI	27 (99/365)
		Henze [60]	2024	100%	CTA FI	37 (118/318)
		**Total**				**33 (237/725)**
	No	Khangure [55][a]	2018	Mixed[b]	CTA FI	31 (23/74)
		Kellomäki [62][c]	2022	0%	CTA FI	22 (56/260)
		**Total**				**24 (79/334)**
	Both	**Total**				**30 (316/1059)**
≥70% stenosis	Yes	Gu [58]	2020	100%	CTA FI	46 (99/215)
		Johansson [59]	2022	100%	CTA+CA FI	55 (63/115)
		Manrique-Zegarra [31][c]	2022	100%	CTA FI	55 (24/44)
		**Total**				**50 (186/374)**
	No	Manrique-Zegarra [31][c]	2022	0%	CA criteria	**57 (4/7)**
	Both	**Total**				**50 (190/381)**
**Restrictive diagnostics**						
≥70% stenosis	No	Ogata [39]	2011	Mixed[b]	CA criteria[d]	10 (34/337)
**Only full collapse**						
≥50% stenosis	Yes	Szabo [145][e]	2001	100%	US Low velocity	17 (10/60)
		Johansson [68]	2015	100%	US Low velocity	2 (5/204)
		**Total**				**6 (15/264)**
	No	Mansour [131]	1995	Mixed[b]	CA "String sign"	5 (12/240)
		Mansour [131]	1995	Mixed[b]	US Low velocity	4 (9/240)
		Anziedei [41]	2009	48%	CA criteria[f]	4 (4/101)
		Khangure [55]	2018	Mixed[b]	US Low velocity	5 (4/74)
		Johansson [56]	2021	77%	US Several[g]	7 (24/351)
		**Total**				**5 (53/1006)**
	Both	**Total**				**5 (68/1270)**
≥70% stenosis	Yes	Szabo [145][e]	2001	100%	US Low velocity	**32 (10/31)**
	No	Anziedei [41]	2009	48%	CA criteria[f]	**5 (4/77)**
	Both	**Total**				**13 (14/108)**

CA: Conventional angiography. CTA: Computed tomography angiography. FI: Feature interpretation. US: Ultrasound.

[a] Some cases overlapping with [68], these were avoided.
[b] Clear that symptomatic and asymptomatic cases were included, but not the share of symptomatic
[c] Presents data for symptomatic and asymptomatic separately. For [62], symptomatic cases were first presented in [58].
[d] Based diagnostics on standard criteria [108] but does not clearly indicate that CNO without full collapse was included.
[e] Higher prevalence might be caused by additional selection (see table 2).
[f] Required an ICA-ratio of <0.2 (except in cases with contralateral occlusion).
[g] Assessed several variants of detecting CNO by low flow velocity and similar, the presented figure is from the definition with highest prevalence: Low flow velocity in the stenosis, possible flow through the stenosis or when suspected despite high flow velocity in the stenosis by low distal velocity (n=2), mismatch between velocity and B-mode (n=3), or very high stenosis pulsatility index (2.73, n=1).

Supplemental table 5 Detailed description of all studies presenting prevalence of CNO in CEA/CAS series

Denominator	≥90% symptomatic	Study	Year	Symptomatic	Diagnostic approach	Prevalence of CNO % (n/N)
**Good diagnostics**						
≥50% stenosis	Yes	NASCET/ECST[a] [110]	2003	100%	CA FI	**10 (262/2718)**
	No	Koskinen [78]	2017	Mixed[b]	CTA criteria	**35 (126/359)**
	Both	**Total**				**13 (388/3077)**
≥70% stenosis	Yes	NASCET/ECST[a] [110]	2003	100%	CA FI	22 (262/1216)
		Zhang [7]	2019	100%	CA criteria	33 (69/210)
		**Total**				**23 (331/1426)**
	No	Ruiz-Salmerón [8]	2013	74%	CA FI	36 (40/111)
		Tsai [6]	2021	47%	CA FI	46 (92/198)
		Yan [3]	2023	63%	CA criteria	19 (20/104)
		**Total**				**37 (152/413)**
	Both	**Total**				**26 (483/1839)**
Performed CEA/CAS	Yes	Zhang [45]	2022	100%	CA criteria	27 (122/444)
		Iner [50][c]	2024	100%	CA criteria	30 (26/88)
		**Total**				**28 (148/532)**
	No	Son [9]	2013	75%	CA criteria	26 (24/91)
		Ohta [2]	2017	56%	CA criteria	21 (21/100)
		İnanç [5]	2020	78%	CA criteria	28 (50/180)
		Štěchovský [10]	2022	45%	CA criteria	13 (84/639)
		Fan [66]	2023	34%	CTA FI	20 (16/79)
		Iner [50][c]	2024	0%	CA criteria	41 (26/64)
		**Total**				**19 (221/1153)**
	Both	**Total**				**22 (369/1685)**
**Restrictive diagnostics**						
≥70% stenosis	Yes	Gonzáles [17]	2011	91%	CA criteria[d]	**16 (116/720)**
	No	Leclerc [141]	1999	Unclear	CA criteria[e]	57 (4/7)
		Oka [30]	2013	67%	CA criteria[d]	20 (10/50)
		**Total**				**25 (14/57)**
	Both	**Total**				**17 (130/777)**
Performed CEA/CAS	Yes	Choi [27]	2010	100%	CA criteria[d]	**29 (48/166)**
	No	Akkan [22]	2018	46%	CA criteria[d]	11 (182/1675)
		Hirata [44]	2014	Mixed[b]	CA criteria[d]	5 (17/345)
		**Total**				**10 (199/2020)**
	Both	**Total**				**11 (247/2186)**
**Only full collapse**						
Performed CEA/CAS	Yes	Meershoek [71]	2018	100%	Mixed[f]	**2 (20/913)**
	No	Berman [135]	1995	Mixed[b]	US Possible flow[g]	10 (26/257)
		Desole [79]	2015	58%	US/CTA mix[h]	2 (33/1414)
		Spacek [14]	2012	58%	CA "string sign"	6 (19/308)
		Omoto [15]	2022	61%	CA "Threadlike"	4 (18/477)
		**Total**				**4 (96/2456)**
	Both	**Total**				**3 (116/3369)**

CA: Conventional angiography. CAS: Carotid artery stenting CEA: Carotid endarterectomy. CTA: Computed tomography angiography. FI: Feature interpretation. US: Ultrasound.

[a] Includes cases randomized to medical arm – but these were selected as eligible for CEA
[b] Clear that symptomatic and asymptomatic cases were included, but not the share of symptomatic
[c] Presents data for symptomatic and asymptomatic separately.
[d] Based diagnostics on standard criteria [108], but added a requirement of [17, 27] or strongly emphasised [22] delayed contrast arrival, or added requirement of ICA-ratio of <0.5 [30], or only presents cases with full collapse while not clearly indicating that CNO without full collapse can exist [44].
[e] Too brief description to be fully assessable (cannot be assessed as "good"), but also not only full collapse by figure example.
[f] Used a mix of modalities and criteria but aimed to only assess cases with full collapse.
[g] Low flow velocity in the stenosis where it is difficult to determine if there is any flow at all.
[h] "String sign" appearance on US colour B-flow and "segmental occlusion" on CTA, i.e. the findings presented in supplemental figure 4C-D.

Supplemental table 6 CNO articles not included in epidemiological assessments

Rationale	Number of articles	References
No denominator	55	1, 4, 11-13, 16, 18-19, 26, 28-29, 32-38, 40, 43, 46-49, 57, 69-70, 72-74, 76, 80, 113, 117, 123, 127, 129, 132, 134, 136, 137, 140, 142-143, 146-156
Reviews, editorials and guidelines	22	106-107, 109, 114, 118, 120-122, 124-126, 144, 157-166
Exams as denominator, i.e. not a patient-centred metric	7	42, 65, 75, 133, 138-139, 167
CNO definition too vague for a reasonable assessment	7	21, 23, 24, 81, 168-170
Duplicates of presented data	16	20, 51-54, 57, 61, 63-64, 67, 108, 130, 171-174
Used ≥60% stenosis (and found 10%, 26/266)	1	87
Used a subset of CA-criteria [108] in a manner that would not exclude conventional stenosis with anatomical variance in the CNO definition as side-to-side ICA difference was sufficient for CNO diagnosis	1	25

Supplemental table 7. Detailed description of management studies that includes a best medical therapy group for symptomatic or a mix of symptomatic and asymptomatic CNO

Study	Design	FC	Diagnosis of CNO	Outcome	Main finding
**Good diagnostics, symptomatic stenosis and IIS outcome – long-term risk**					
NASCET/ECST [108, 110]	RCT. 148 CEA+BMT Vs 114 BMT	6%[a]	FI of CA	5-year IIS + PO	≈ 16.8% Vs. ≈ 15.1% ARR -1.7%, p=0.90[b]
Garciá-Pastor [34]	Observational. 70 CEA/CAS + BMT Vs 71 BMT	36%[a]	CA criteria	2-year IIS after PE + PO	10.2% Vs. 12.0%, p=0.82[b]
**Good diagnostics, symptomatic stenosis and IIS outcome – short-term risk before/without CEA/CAS**					
Garciá-Pastor [35]	Observational. 41 FC BMT Vs 73 Not FC BMT	36%[a]	CA criteria	90-day IIS after PE[c]	0% Vs 6%, p=0.15
Johansson [57]	Observational. 46 FC BMT Vs 70 Not FC BMT	40%[d]	FI of CTA	2-day and 28-day IIS after PE[c]	2 days: 22% Vs 3% 28 days: 36% Vs 3%, p<0.001
Henze [60]	Observational. 26 FC BMT Vs 92 Not FC BMT	22%[e]	FI of CTA	2-day and 28-day IIS after PE[c]	2d: 16% Vs 3%, p=0.01 28d: 22% Vs 16%, p=0.22
**Only full collapse, symptomatic stenosis and IIS outcome – long-term risk**					
O'Leary [113]	Observational 23 CEA+BMT Vs 9 BMT	100%[a]	CA threadlike	IIS during mean 34 month follow-up + PO	9% Vs 33%[f]
**Other: Not symptomatic stenosis, not IIS outcome and/or too inclusive CNO definition.**					
Ogata [39]	Observational. 34 CNO Vs 303 CS. Mix of BMT/CEA/CAS and symptomatic/ asymptomatic	Unclear	Restrictive CA criteria[g]	5-year risk of any stroke	12% vs 8%, p=0.43.
Song [12]	Observational. 48 CAS+BMT Vs 31 BMT, only symptomatic	Unclear	Restrictive CA criteria[g]	1-year IIS or TIA + PO	2% Vs 19% p=0.026
Radak [80]	Observational. 259 CEA+BMT vs 50 BMT, only symptomatic	Unclear	Mixed, most likely included CS in CNO group[h]	1-year IIS + PO	2% Vs 14%, p<0.001

BMT: Best Medical treatment. CAS: Carotid artery stenting. CEA: Carotid endarterectomy. CNO: Carotid near-occlusion. CS: Conventional stenosis. ECST: European Carotid Stenosis Trial. FC: Full collapse. IIS: Ipsilateral ischemic stroke. MACE: Stroke, Myocardial infarction or death. NASCET: North American symptomatic carotid endarterectomy trial. PE: Presenting event. PO: Perioperative risk (30-day risk of stroke or death)

There are several additional articles of these materials, with either duplicate data or shorter follow-up [33, 58, 68, 174].
Ringelstein et al [133] is known to many as an indicator of treatment for CNO but does not actually present any outcomes.
Ogata not presented in table 3 as it mixed symptomatic and asymptomatic patients.

Additional design issues: Two were quite selective (14-28% had BMT alone) [80, 113]. One assessed events as a secondary outcome (focus was cognition), presented few details about how events were registered [12]. One used no statistics and varying follow-up time [113]. One clearly included patients with conventional stenosis in the CNO group [80].

[a] Full collapse defined by appearance.
[b] No events among CNO with full collapse. ARR was numerically presented, the risk figures were derived from reading the published Kaplan-Meier curves at 5 years.
[c] Counted before CEA/CAS or until end of follow-up for those not yet treated.
[d] Creates a prognostic definition of full collapse: Distal ICA diameter ≤2.0 mm and/or ICA ratio ≤0.42.
[e] Prespecified definition of full collapse as distal ICA diameter ≤2.0 mm and/or ICA ratio ≤0.42 [57]
[f] Not statistically evaluated. As follow-up time seemed to vary widely, χ^2-test is inappropriate to apply for this review.
[g] Based diagnostics on standard criteria [108] but does not clearly indicate that CNO without full collapse was included.
[h] Either obvious distal ICA diameter reduction on non-specified angiography or stenosis PSV ≥2.3 m/s, stenosis EDV ≥1.0 m/s and ICA/CCA velocity ratio (likely of PSV) of >4. These thresholds had no reference and seemed arbitrary as no relevant US study existed at the time. Only a subset of cases underwent angiography. While these diagnostic thresholds have never been formally assessed, they are near or below the mean values of conventional ≥50% stenosis presented in a later study: 289 cm/s, 94 cm/s and 4.4 respectively [56].

Supplemental table 8. Perioperative risk and long-term follow-up after CEA/CAS

Method	Study	Symptomatic	Diagnostic approach	Full collapse	30-day risk of stroke or death	Annual stroke risk post-op
**Good diagnostics**						
CEA	NASCET/ECST [110]	100%	CA FI	6%	5.4% (8/148)	2.2% (14/639)[a]
	Garciá-Pastor [36]	100%	CA criteria	24%	13.0% (3/23)	2.3% (1/44)
	Yan [11]	92%	CA criteria	27%	2.6% (1/38)	1.2% (1/87)
	Kim [37]	100%	CA criteria	56%	3.6% (1/28)	-
	Gu [58][c]	100%	CTA FI	19%	3.7% (2/54)	-
	Zhang [45]	100%	CA criteria	44%	0.8% (1/122)	0.0% (0/112)
	**Total**				**3.8% (16/413)**	**1.8% (16/879)**
CAS	Tsai [6]	47%	CA FI	49%	1.1% (1/92)	-
	Son [9]	75%	CA criteria	Unclear[c]	4.2% (1/24)	0.0% (0/26)
	Yan [11]	96%	CA criteria	28%	0.0% (0/57)	0.8% (1/123)
	Ruiz-Salmerón [8]	74%	CA criteria	Unclear[c]	9.3% (5/54)	-
	İnanç [5]	78%	CA criteria	Unclear[c]	6.0% (3/50)	-
	Štěchovský [10]	45%	CA criteria	Unclear[c]	8.3% (7/84)	-
	García-Pastor [36]	100%	CA criteria	33%	2.3% (1/44)	1.2% (1/84)
	Sakamoto [18]	71%	CA CCA-ratio[d]	Unclear[c]	0.0% (0/14)	0.0% (0/18)
	Kim [37]	100%	CA criteria	43%	5.9% (1/17)	-
	Cay [4]	71%	CA criteria	19%	3.4% (2/58)	-
	**Total**				**4.3% (21/494)**	**0.8% (2/251)**
**Restrictive diagnostics**						
CEA	No studies				-	-
CAS	González [17]	91%	CA criteria[e]	29%	0.0% (0/116)	0.3% (1/339)
	Oka [30]	67%	CA criteria[e]	Unclear[f]	6.7% (1/15)	0.0% (0/49)
	Matsuda [19]	75%	CA criteria[e]	Unclear[f]	5.4% (3/56)	-
	Song [12]	100%	CA criteria[e]	Unclear[f]	0.0% (0/48)	0.0% (0/44)
	**Total**				**1.8% (4/235)**	**0.2% (1/432)**
**Only full collapse**						
CEA	O'Leary [113]	92%	CA Threadlike	100%	8.0% (2/25)	0.0% (0/69)
	Fredricks [134]	71%	CA Threadlike	100%	3.6% (1/28)	-
	Kniemeyer [148]	84%	CA Threadlike	100%	9.9% (8/81)	-
	Ascher [143]	Unclear	US Low flow	100%	0.0% (0/12)	-
	Greiner [147]	100%	CA Threadlike	100%	10.5% (4/38)	0.0% (0/149)
	Desole [79]	58%	US/CTA mix[g]	100%	0.0% (0/33)	0.0% (0/96)
	Meershoek [71]	100%	Mixed[h]	100%	5.9% (1/17)	0.0% (0/31)
	Pagliaricco [69][d]	29%	CTA criteria[i]	100%	7.1% (2/28)	0.0% (0/26)
	**Total**				**6.9% (18/262)**	**0.0% (0/371)**
CAS	Nikas [16]	80%	CA Threadlike	100%	0.0% (0/25)	0.0% (0/23)
	Terada [13]	85%	CA Threadlike	100%	0.0% (0/20)	0.0% (0/40)
	Spacek [14]	58%	CA Threadlike	100%	7.7% (1/13)	0.0% (0/5)
	Neves [1]	100%	CA Threadlike	100%	4.2% (1/24)	1.6% (2/126)
	Omoto [15]	61%	CA Threadlike	100%	0.0% (0/18)	-
	**Total**				**2.0% (2/100)**	**1.0% (2/194)**

CA: Conventional angiography. CAS: Carotid artery stenting CEA: Carotid endarterectomy. CTA: Computed tomography angiography. FI: Feature interpretation. US: Ultrasound

Annual stroke risk post-op: Defined in supplemental table 9.
CNO Studies with similar outcome that were not included:

- Likely included conventional stenosis in the CNO definition or CNO is described too poorly to be assessable [21, 25, 29, 42, 80, 127, 136, 168-170]
- Other or poorly described outcomes [2, 7, 22, 27, 39, 44, 50, 140]
- <10 cases [23, 26, 28, 48, 68, 76, 133]
- Staged CAS [24]
- Duplicate data [61, 171].
- One previous meta-analysis [121] included a conference abstract (Razuk A, Caffaro R, Karakahanian W, et al. Carotid String Sign: Is There a Role for Carotid Stenting? 38th Annual Symposium of Society for Clinical

Vascular Surgery Arizona, USA, 2010). These findings have not been presented in a peer-review journal, nor have we been able to locate that abstract. Supposedly, the study included 13 CAS-treated patients.

[a] Actual numbers not reported, why we made estimates: 5-year risk assessed by reading the surgical groups Kaplan-Meier curve at 5 years (Figure 4, middle column, top row in [110]: 15.1%. With 148 subjects, 22 events reasonably occurred. This curve also includes perioperative risk, where 8 events occurred (36% of events). Thus, 14 events were estimated during post-op period (64% of events). Follow-up time was estimated from same figure. We summed the number of patients left in the analysis at year 1, 2, 3, 4 and 5 and added half of each year's drop-out (thus assuming drop-outs occurred evenly through the year that passed).
[b] One case underwent CAS without complications.
[c] Clear that both CNO with and without full collapse were included, but number with full collapse not reported.
[d] Distal ICA/CCA ratio. <0.42 for all [13] or <0.40 for men and <0.45 for women [18]
[e] Based diagnostics on standard criteria [108], but added a requirement of delayed contrast arrival [17], added requirement of ICA-ratio of <0.5 [30], or only presents cases with full collapse while not clearly indicating that CNO without full collapse can exist [12, 19].
[f] Clear that CNO with full collapse were included, but not certain that CNO without full collapse were.
[g] "String sign" appearance on colour B-flow and "segmental occlusion" on CTA, i.e. the findings presented in supplemental figure 7C-D.
[h] Used a mix of modalities and criteria but aimed to only assess cases with full collapse.
[i] Presents data for with and without full collapse, but we only use the full collapse data: Defines full collapse by partially using a suggest definition of full collapse (only <2.0 mm distal ICA, not also the "and/or ICA ratio ≤0.42") [57], resulting in that some with full collapse were missed, but not vice-versa. Without full collapse was not used as there was no CNO definition or diagnostic description of how CNO was diagnosed.

Supplemental table 9. Summary of perioperative risk and long-term follow-up after CEA/CAS based on symptomatic status, full collapse status and timing in full collapse.

	Studies	30-day risk stroke or death	Annual stroke risk post-op
**CEA studies**			
Symptomatic stenosis	36-37, 45, 58, 71, 117, 139	4.7% (20/430)	1.5% (15/975)
Asymptomatic stenosis	No studies	No studies	No studies
Full collapse (symptomatic or asymptomatic)	45, 58, 69, 71, 79, 110, 124, 134, 139-140	6.4% (21/326)	0.0% (0/421)
Without full collapse (symptomatic or asymptomatic)[a]	45, 58, 117	3.1% (8/259)	2.0% (14/701)
Symptomatic full collapse	45, 58, 71, 139	6.7% (8/119)	0.0% (0/230)
Symptomatic without full collapse[a]	45, 58, 117	3.1% (8/259)	2.0% (14/701)
Symptomatic full collapse, treatment within 48 hours.	No studies	No studies	No studies
All studies	11, 36-37, 45, 58, 69, 71, 79, 117, 110, 124, 134, 139-140	5.0% (34/675)	1.3% (16/1253)
**CAS studies**			
Symptomatic stenosis	1, 4, 10-12, 36-37	4.2% (9/212)	1.2% (3/254)
Asymptomatic stenosis	4, 10	4.8% (3/63)	No studies
Full collapse (symptomatic or asymptomatic)	1, 6, 13-17, 30	2.1% (4/193)	0.8% (2/241)
Without full collapse (symptomatic or asymptomatic)	6, 13, 17	0.0% (0/163)	No studies[b]
Symptomatic full collapse	1	4.2% (1/24)	1.6% (2/126)
Symptomatic without full collapse	No studies	No studies	No studies
Symptomatic full collapse, treatment within 48 hours.	No studies	No studies	No studies
All studies	1, 4-6, 8-19, 30, 36-37	3.3% (27/826)	0.6% (5/877)
**Mixed CEA and CAS**			
Symptomatic full collapse	36	6.7% (1/15)	No studies
Symptomatic not full collapse	36	8.3% (3/36)	No studies

CAS: Carotid artery stenting CEA: Carotid endarterectomy.

Forest plots are presented in supplemental figure 1-4.

Annual stroke risk post-op: Annual risk of ipsilateral ischemic stroke from day 31 until end of follow-up. Calculated by dividing number of events during follow-up with patient-years of follow-up. Thus, the denominator is not patients but patient-years. Patient-years of follow-up estimated as mean/median follow-up time times number of patients with follow-up data. When data was not presented in numbers, it was extracted from figures when needed (specified in supplementary table 8).

Except for NASCET/ECST[a], studies were selected if they only included cases with the selection criteria presented or presented data for the categories separately.

[a] All cases in NASCET/ECST included, given 94% without full collapse and no events in full collapse group.
[b] Only a single patient assessed with no events during 2-year follow-up [13].

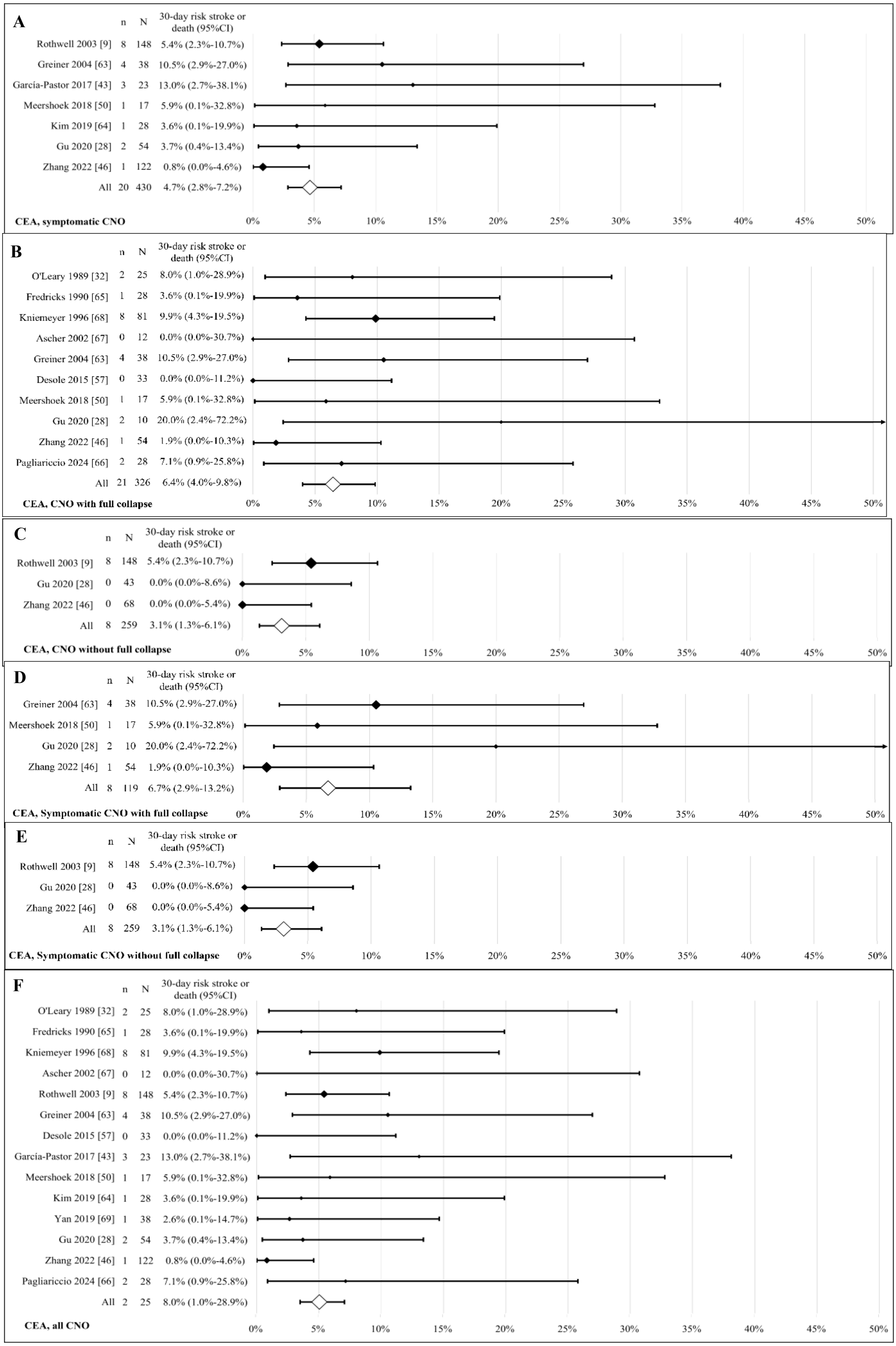

Supplemental figure 1. 30-day risk of stroke or death after CEA. A) Symptomatic CNO. B) CNO with full collapse. C) CNO without full collapse. D) Symptomatic CNO with full collapse. E) Symptomatic CNO without full collapse F) All CNOs. C and E contain the same studies.

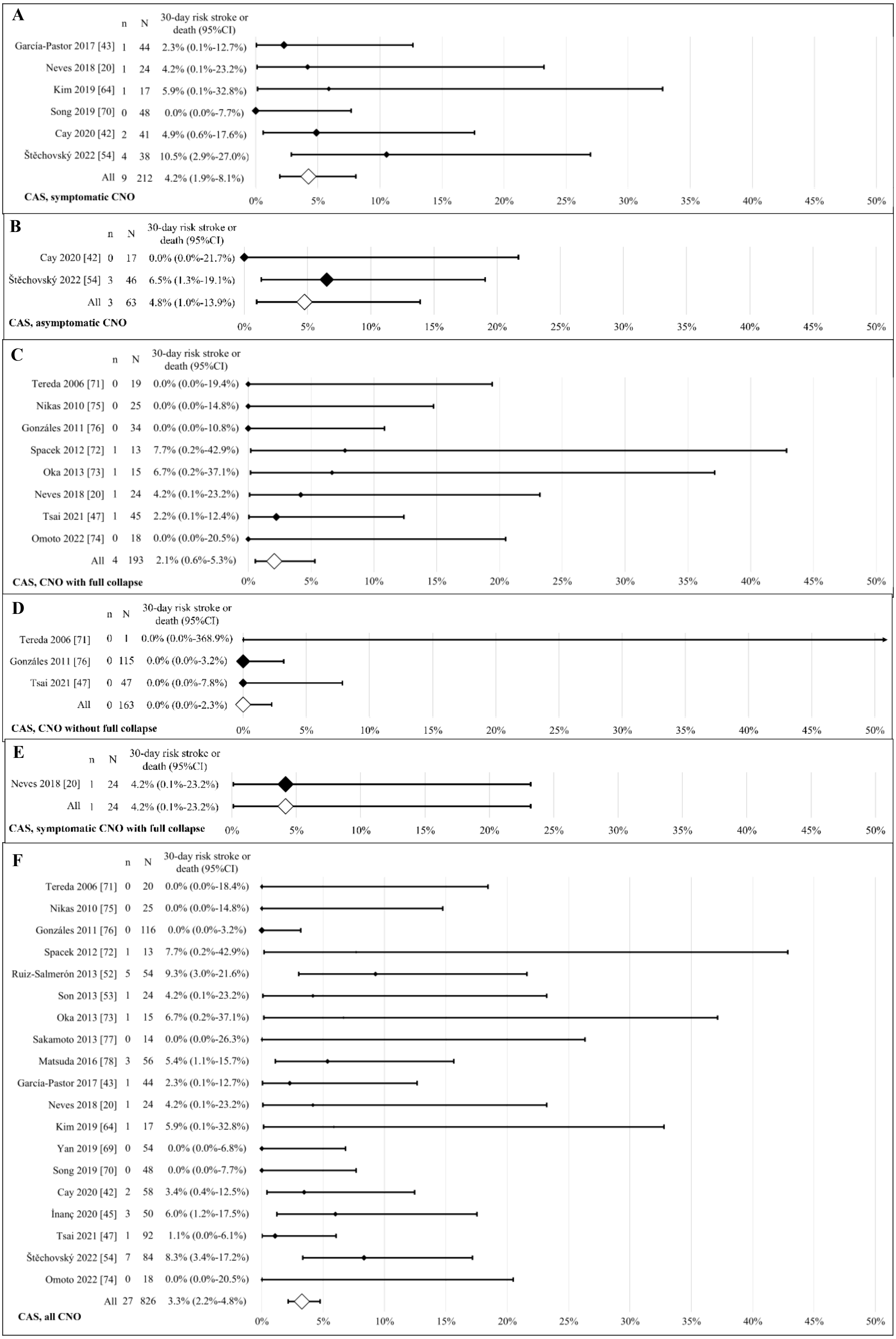

Supplemental figure 2. 30-day risk of stroke or death after CAS. A) Symptomatic CNO. B) Asymptomatic CNO. C) CNO with full collapse. D) CNO without full collapse. E) Symptomatic CNO with full collapse. F) All CNOs.

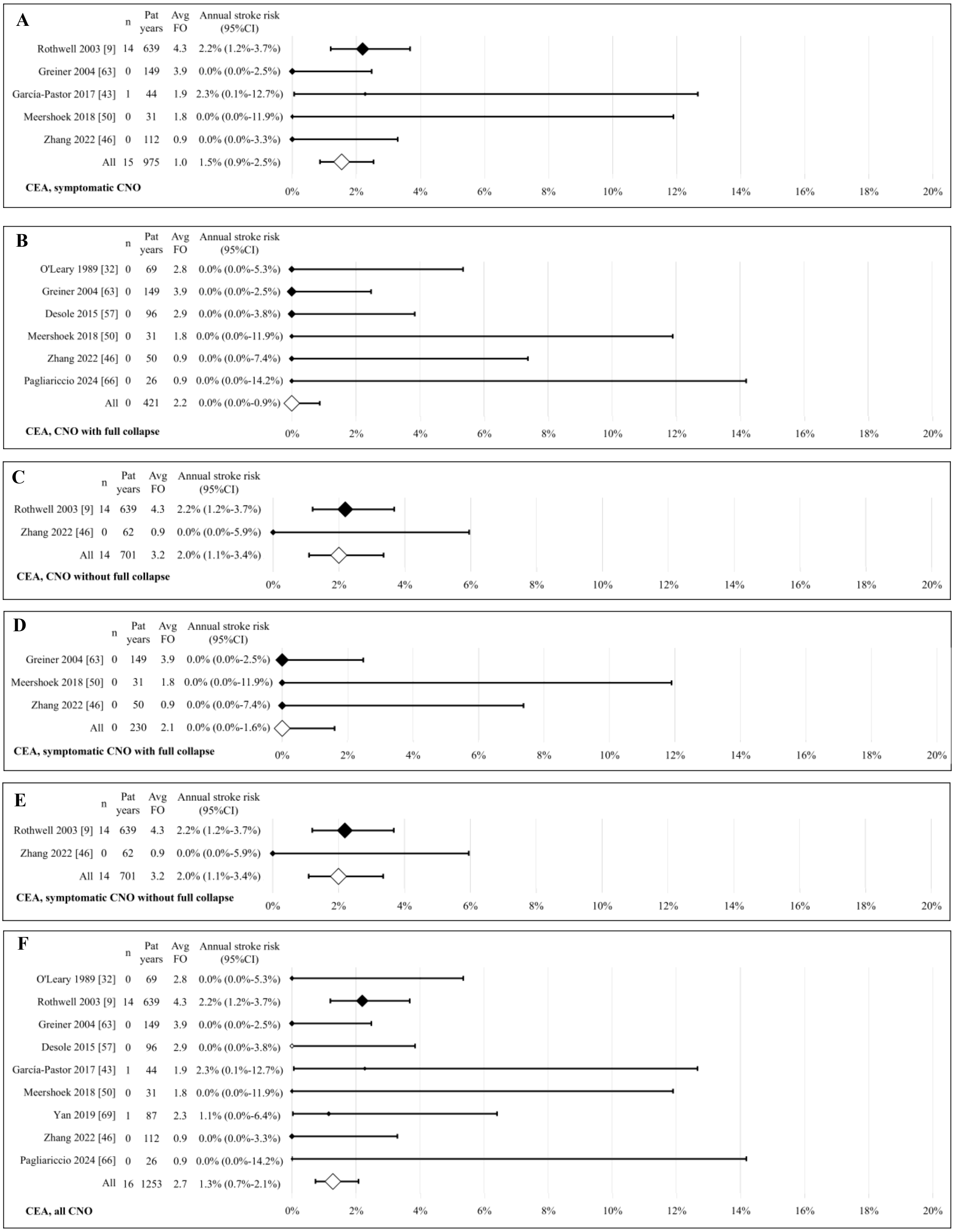

Supplemental figure 3. Annual risk of ipsilateral ischemic stroke after CEA. Patient years (“Pat years”) calculated as mean or median follow-up (“Avg FO”) from day 31 times number of patients. A) Symptomatic CNO. B) CNO with full collapse. C) CNO without full collapse. D) Symptomatic CNO with full collapse. E) Symptomatic CNO without full collapse F) All CNOs.

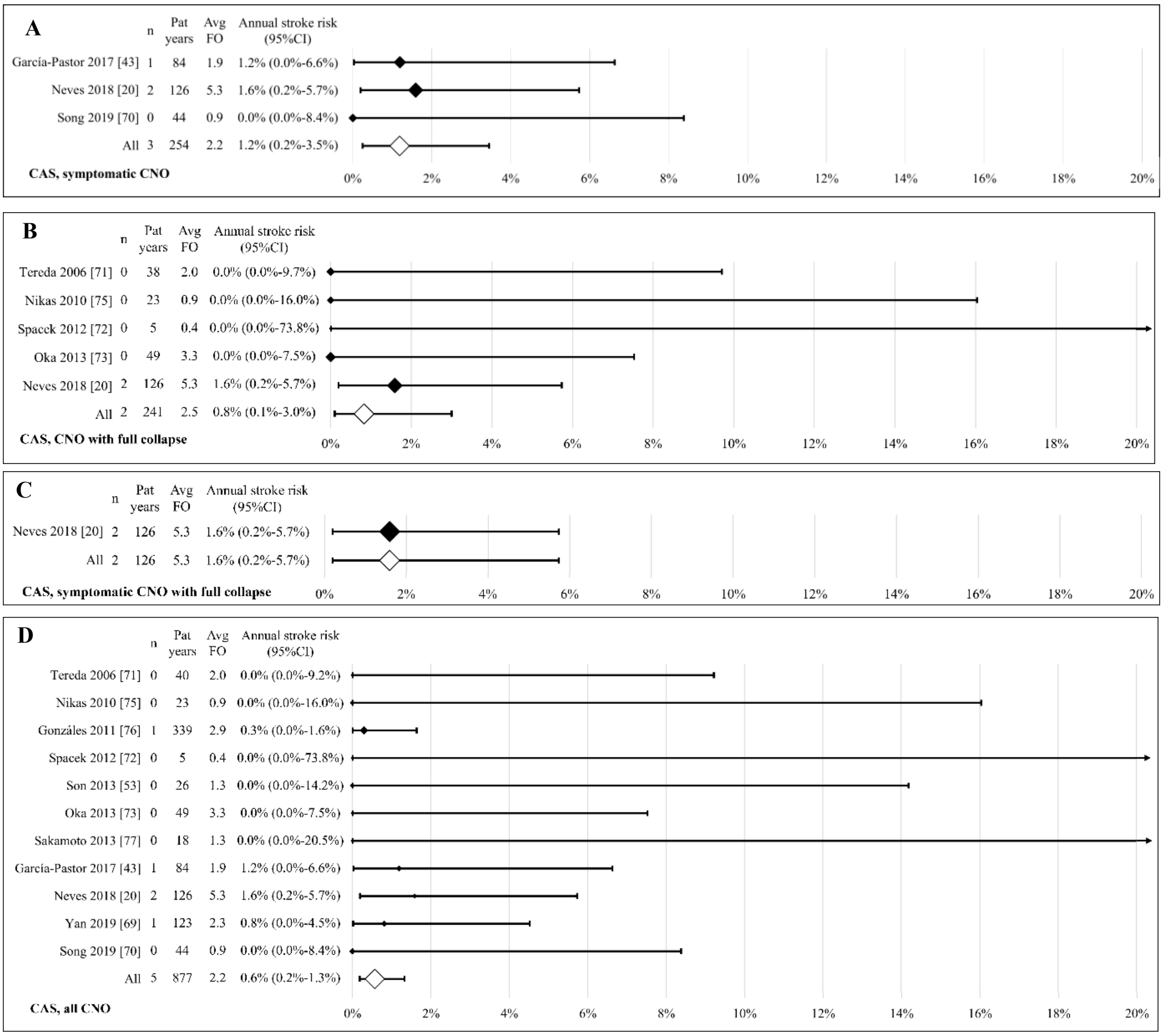

Supplemental figure 4. Annual risk of ipsilateral ischemic stroke after CEA. Patient years ("Pat years") calculated as mean or median follow-up ("Avg FO") from day 31 times number of patients. A) Symptomatic CNO. B) CNO with full collapse. C) Symptomatic CNO with full collapse. D) All CNOs.

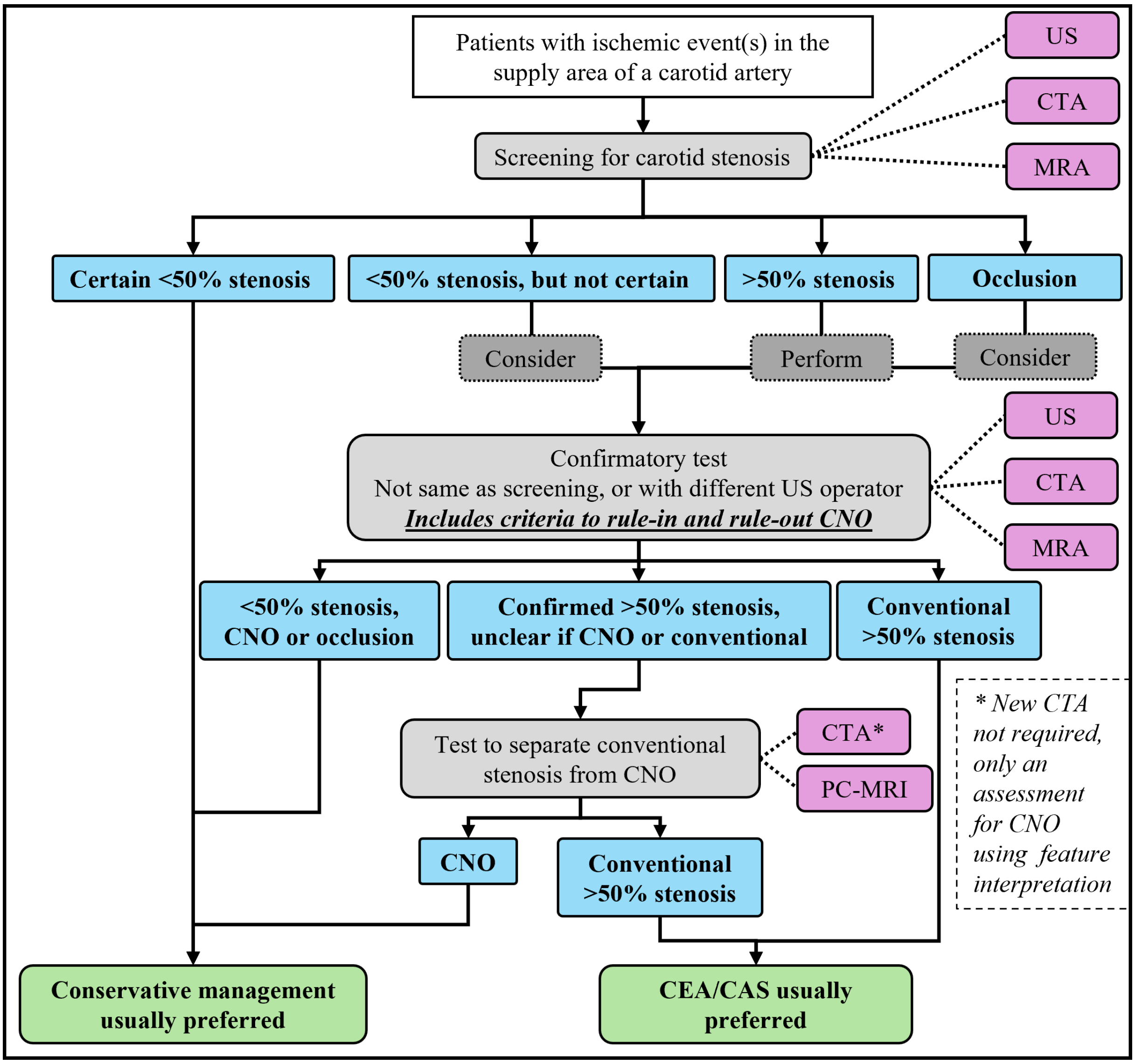

Supplemental figure 5. Alternative future diagnostic pathway for carotid stenosis. The concept is the same as in figure 3, except for the introduction of pair of criteria that are very sensitive (can rule-out) or very specific (can rule-in) CNO. No such validated criteria exist (studies creating and validating them are warranted). These novel criteria should be based on feasible methods, such as US velocity och CTA measurements. The central difference from current approaches is that a threshold will only be either sensitive or very specific (not both at the same time), and there is to be one of each. Hence, the intention is to find cases where CNO status is still unclear after using feasible methods and only subject these cases to more advanced methods. CNO: Carotid near-occlusion. CTA: Computed tomography angiography. MRA: Magnetic resonance angiography (i.e. traditional lumen assessment, preferably contrast-enhanced). PC-MRI: Phase-contrast magnetic resonance imaging. US: Ultrasound.

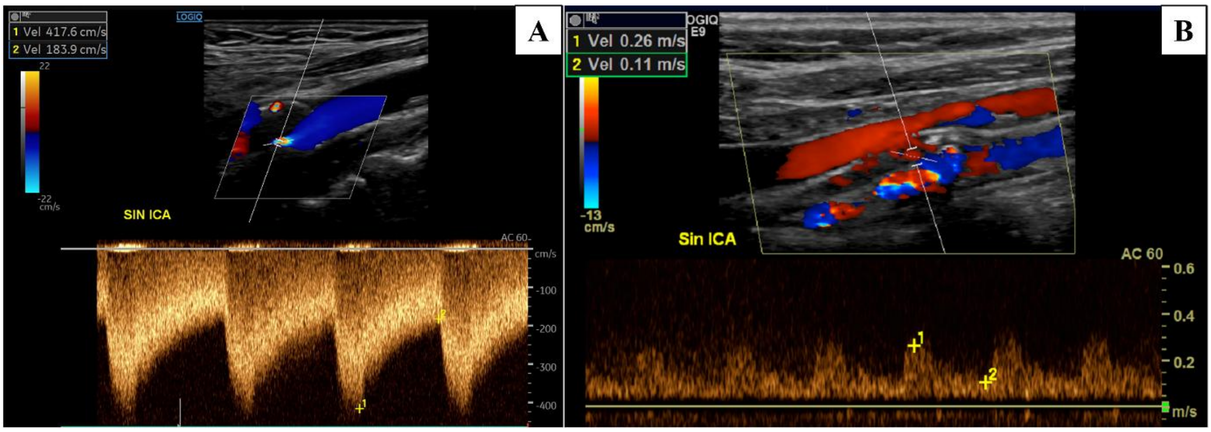

Supplemental figure 6: 2 cases of left-sided CNO, ultrasound findings. A) A severe stenosis with high flow velocity in the stenosis. The typical finding in CNO, seen in almost all CNO without full collapse and many CNO with full collapse. Not distinguishable from conventional stenoses. B) A severe stenosis with low flow velocity in the stenosis. Very specific for CNO and indicates full collapse.

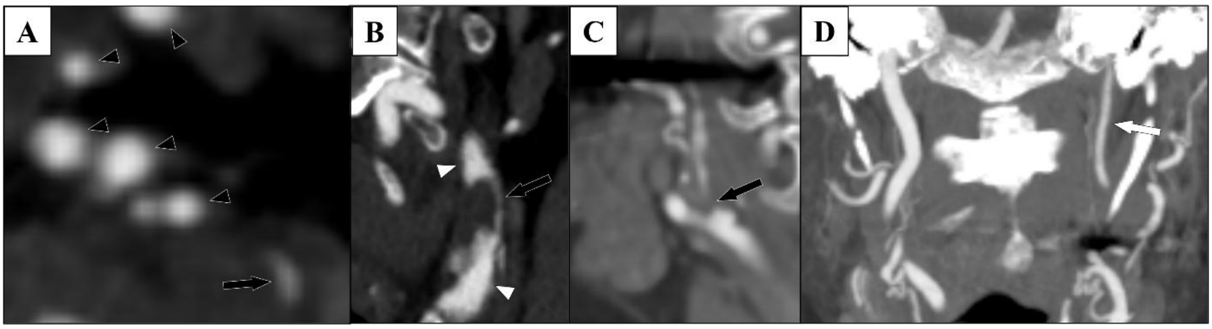

Supplemental figure 7: Partial volume effect on CTA. A+B) Same case as figure 1B. A) Axial view of a severe stenosis (black arrow) which is darker than several ECA branches (black arrowheads). B) Coronal view, the stenosis (black arrow) is also darker than the ICA proximal and distal (white arrowheads). The stenosis lumen is smaller than the voxels. Bright contrast and dark tissue is mixed and only the mean value is presented, which results in a darker appearance. This is seen in 49% of CNOs [56]. C+D) Is another case. C) Sagittal view of a very severe stenosis where no contrast is visible for a short segment (black arrow). D) The distal ICA (white arrow) is clearly contrast filled. The most reasonable explanation is that the partial volume effect is so severe that the existing contrast in the stenosis is not visible – but can be presumed from context (seen more directly more distally in C and very clearly in D). The alternative is that this is an occlusion, and the distal ICA is filled retrograde from the skull. This alternative is unreasonable as without an outflow, it should not be possible for contrast to mix into a long stump, let alone mix well already during the arterial phase. 8% of CNOs are mistake for occlusion when assessed in routine practice due to this phenomenon [14]. This has even been called “segmental occlusion” in the literature [57], a notion we disagree with: One needs to add logic to the contrast, not just assess what is visible.

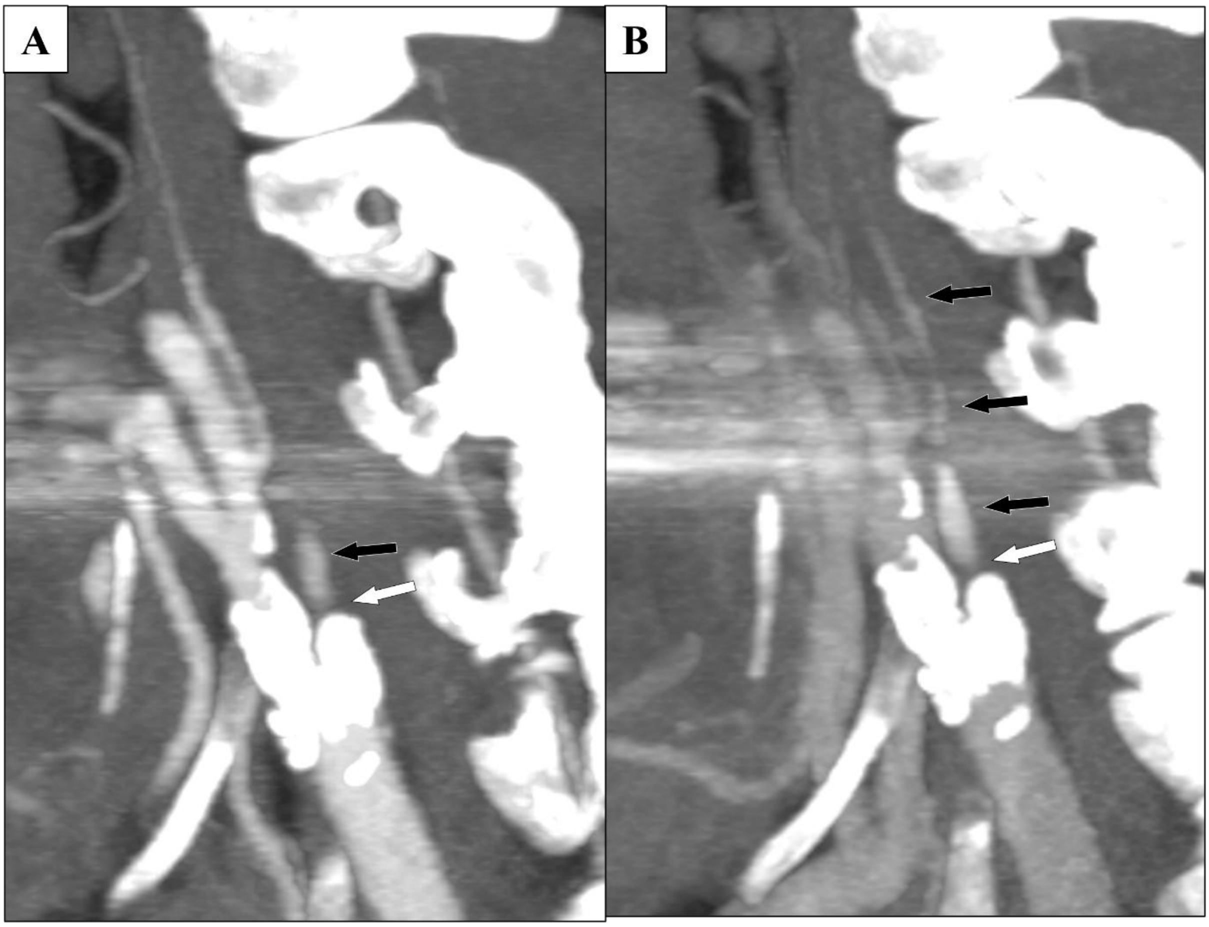

Supplemental figure 8: Severe variant of CNO with full collapse examined with multiphase CTA, sagittal views. A) Arterial phase. After a severe stenosis where contrast is not visible (partial volume effect, white arrow), contrast is seen again in short segment (black arrow). B) Venous phase, 6 seconds later. The contrast has progressed 2.1 cm more distally (black arrows), resulting in a flow velocity of 0.35 cm/s (<1% of normal). This phenomenon, where contrast has not reached the skull base at time of image capture, has been known among experts, but not clearly described previously and is very easy to mistake for occlusion. CTA is not an examination of arteries but an exam of where contrast is at the time of image capture. Three minor aspects: 1) A severe distal stenosis or intracranial occlusion cannot be excluded. 2) Even though flow is proven, contrast is still not seen in the most severe stenosis (white arrow), which is expected by the notion of partial volume effect. 3) A flow phenomenon is seen in the contrast beyond the stenosis compared to surrounding arteries: It is darker in arterial phase (not reached yet) and brighter in the venous phase (not washed out yet), similar to the arterial phase of panel A (windowing is the same).
